# Interplay between Sulfur Assimilation and Biodesulfurization Activity in Rhodococcus qingshengii IGTS8: Insights into a Regulatory Role of the Reverse Transsulfuration Pathway

**DOI:** 10.1128/mbio.00754-22

**Published:** 2022-07-20

**Authors:** Olga Martzoukou, Panayiotis D. Glekas, Margaritis Avgeris, Diomi Mamma, Andreas Scorilas, Dimitris Kekos, Sotiris Amillis, Dimitris G. Hatzinikolaou

**Affiliations:** a Enzyme and Microbial Biotechnology Unit, Department of Biology, National and Kapodistrian University of Athens, Athens, Greece; b Sector of Biochemistry and Molecular Biology, Department of Biology, National and Kapodistrian University of Athens, Athens, Greece; c Laboratory of Clinical Biochemistry—Molecular Diagnostics, Second Department of Pediatrics, School of Medicine, National and Kapodistrian University of Athens, P. & A. Kyriakou Children's Hospital, Athens, Greece; d Biotechnology Laboratory, Sector of Synthesis and Development of Industrial Processes (IV), School of Chemical Engineering, National Technical University of Athens, Athens, Greece; University of Delaware

**Keywords:** biocatalysis, genetic engineering, reverse transsulfuration, sulfur metabolism, cysteine, cystathionine, methionine, cystathionine β-synthase, cystathionine γ-lyase

## Abstract

Biodesulfurization is a process that selectively removes sulfur from dibenzothiophene and its derivatives. Several natural biocatalysts harboring the highly conserved desulfurization operon *dszABC*, which is significantly repressed by methionine, cysteine, and inorganic sulfate, have been isolated. However, the available information on the metabolic regulation of gene expression is still limited. In this study, scarless knockouts of the reverse transsulfuration pathway enzyme genes *cbs* and *metB* were constructed in the desulfurizing strain *Rhodococcus* sp. strain IGTS8. We provide sequence analyses and report the enzymes’ involvement in the sulfate- and methionine-dependent repression of biodesulfurization activity. Sulfate addition in the bacterial culture did not repress the desulfurization activity of the Δ*cbs* strain, whereas deletion of *metB* promoted a significant biodesulfurization activity for sulfate-based growth and an even higher desulfurization activity for methionine-grown cells. In contrast, growth on cysteine completely repressed the desulfurization activity of all strains. Transcript level comparison uncovered a positive effect of *cbs* and *metB* gene deletions on *dsz* gene expression in the presence of sulfate and methionine, but not cysteine, offering insights into a critical role of cystathionine β-synthase (CβS) and MetB in desulfurization activity regulation.

## INTRODUCTION

Microbial elimination of dibenzothiophene (DBT) and related organosulfur compounds could allow the biodesulfurization of oil products by selectively removing sulfur from carbon-sulfur bonds, thus maintaining the calorific value of the fuel (1, 2). The process is mediated by the well-characterized 4S metabolic pathway that is found in several genera, with the most prominent being that of rhodococci (3). The three biodesulfurization genes are organized in a plasmid-borne operon, *dszABC*, and encode a DBT-sulfone monooxygenase (*dszA*), a 2-hydroxybiphenyl-2-sulfinate (HBPS) desulfinase (*dszB*), and a DBT monooxygenase (*dszC*), respectively. A fourth chromosomal gene, designated *dszD*, encodes an NADH-FMN reductase that energetically supports the pathway. One of the major disadvantages in exploiting the biotechnological potential of the biodesulfurization process is the sulfate-, methionine-, and cysteine-mediated transcriptional repression of *dsz* genes through a putative repressor-binding site in the *P_dsz_* promoter. The operon is derepressed in the presence of organosulfur compounds such as DBT and dimethyl sulfoxide (DMSO), and Dsz enzymes are considered sulfate starvation-induced (SSI) proteins (4–6).

**FIG 1:**
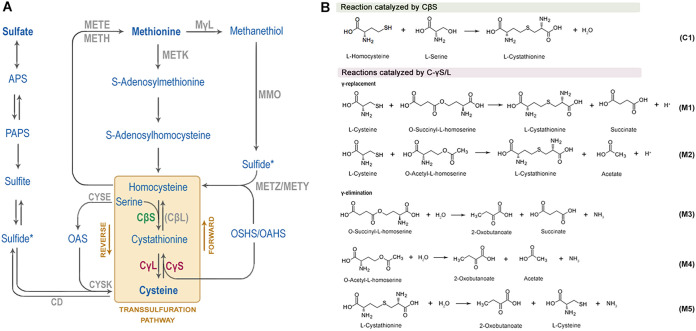
Bacterial sulfur metabolism. (A) Overview of standard methionine and cysteine biosynthesis and interconversion routes in bacteria as part of the sulfur assimilation pathway (APS, adenylylsulfate; PAPS, 3′-phosphoadenylyl sulfate; OAS, *O*-acetyl-l-serine; OSHS, *O*-succinyl-l-homoserine; OAHS, *O*-acetyl-l-homoserine). Asterisks indicate pathway interconnection points. (B) Canonical reactions of sulfur metabolism catalyzed by CβS and MetB (C-γS/L) in the order *Corynebacteriales*.

Mechanistic insight into the molecular regulation of the *dsz* operon was gained recently with the identification of the TetR family activator DszGR and the WhiB1 repressor, both derived from the desulfurizing bacterium *Gordonia* sp. strain IITR100 (6–8). Binding of DszGR to the promoter DNA induces an initial bend in the *Gordonia* sp. *P_dsz_* region but requires the integration host factor (IHF), which in turn plays a major role in promoter activity (9). Despite the high homology between *dszABC* operons of Gordonia alkanivorans RIPI90A and *Rhodococcus* strain IGTS8, promoter sequences are only partially conserved (10). Moreover, a DszR activator also facilitated by the IHF was reported for the activation of a σN-dependent *dsz* promoter by metagenomic functional analysis (11). However, information on the global regulation of sulfur metabolism is still limited, and the sulfur assimilation pathways of *Rhodococci* had been investigated only *in silico* (12, 13). An exception is a very recent study that conducted comparative proteomics and untargeted metabolomics analyses in Rhodococcus qingshengii IGTS8 and proposed a working model for assimilatory sulfur metabolism reprogramming in the presence of DBT (4). Moreover, the effects of carbon and sulfur source on biotransformation of 6:2 fluorotelomer sulfonic acid were examined in Rhodococcus jostii RHA1 (14). General aspects of carbon and nitrogen metabolism of oleaginous *Rhodococcus* spp. were elucidated, due to the ability of this rhodococcal group (Rhodococcus opacus, *R. jostii*, R. wratislaviensis, and R. imtechensis) to synthesize and accumulate specific lipids of biotechnological interest (15). However, knowledge concerning sulfur metabolism and especially methionine-cysteine interconversion routes in *Rhodococcus* and other desulfurizing species is still relatively limited.

l-Methionine and l-cysteine, the sulfur-containing amino acids responsible for *dsz* repression, are interconverted with the intermediary formation of l-homocysteine and l-cystathionine through the transsulfuration metabolic pathway. l-Methionine can be converted to l-homocysteine via two possible routes (Fig. 1A). The first requires the catalytic action of a methionine γ-lyase (MγL) for methanethiol production (16), which is then oxidized to sulfide by a methyl mercaptan oxidase (MMO) present in *Rhodococcus* strain IGTS8 (17). A direct sulfhydrylation pathway can convert sulfide to l-homocysteine, in condensation with either *O*-succinyl-l-homoserine (OSHS) or *O*-acetyl-l-homoserine (OAHS), through the catalytic action of MetZ or MetY, respectively (4, 18). A second pathway for methionine catabolism involves the sequential formation of *S*-adenosyl-l-methionine (SAM), *S*-adenosyl-l-homocysteine (SAH), and l-homocysteine (4, 19–23). In the first step of the forward transsulfuration pathway, a γ-replacement reaction of l-cysteine and an activated l-homoserine ester (OSHS/OAHS) generates l-cystathionine, with the catalytic action of a cystathionine γ-synthase (CγS) (Fig. 1B, reactions M1 and M2) (24, 25). In the second forward transsulfuration step, l-cystathionine is acted upon by a cystathionine beta-lyase (CβL) to form l-homocysteine. This in turn, can be converted to l-methionine through a methylation step or serve as the precursor for l-cysteine biosynthesis via the reverse transsulfuration pathway (25). Therein, a cystathionine β-synthase (CβS)-mediated condensation of l-homocysteine with l-serine generates l-cystathionine (Fig. 1B, reaction C1), which is then cleaved by a cystathionine γ-lyase (CγL) to form l-cysteine (Fig. 1B, reaction M5). Both key enzymes of the reverse transsulfuration pathway, CβS and CγL, are pyridoxal phosphate (PLP) dependent (26–29).

This reverse transsulfuration metabolic route was reported in mammals, yeasts, archaea, and several bacteria (20, 30–34). Alternative biocatalytic reactions of CβS which generate H_2_S in the presence of cysteine were reported in bacterial species and eukaryotic cells. Interestingly, a condensation of cysteine with homocysteine catalyzed by CβS can produce cystathionine (35, 36). However, H_2_S production was studied recently in knockout strains of Mycobacterium tuberculosis, a species closely related to *R. qingshengii* IGTS8. Therein, the cysteine desulfhydrase Cds1, but not CβS, was shown to be responsible for these reactions (37). Another pathway for l-cysteine biosynthesis requires the *O*-acetyl-l-serine (OAS) sulfhydrylase CysK for the condensation of sulfide and OAS (4, 38). In the opposite direction, a reaction mediated by l-cysteine desulfhydrase (CD) leads to l-cysteine degradation to sulfide, pyruvate, and ammonia (38).

The genome of the model biocatalyst *R. qingshengii* IGTS8, harbors genes for CβS and cystathionine γ-synthase/lyase (C-γS/L), an indication for an active reverse transsulfuration pathway. The gene product of *cbs* is annotated as a putative CβS Rv1077, whereas *metB* is predicted to encode a C-γS/L. Transposon-mediated disruption of the *cbs* gene was reported in the desulfurizing strain Rhodococcus erythropolis KA2-5-1, and it was suggested that sulfate and methionine are indirectly involved in the repression of the *dsz* phenotype (39). However, sulfur assimilation pathways and the regulation of *dsz* expression in response to different sulfur sources in desulfurizing *Rhodococcus* species remains largely understudied *in vivo*.

Several genetic modifications were conducted with a direct biotechnological approach, aiming to increase the efficiency of biodesulfurization rather than elucidate the underlying sulfur assimilation regulatory mechanisms. As such, most of them engineer Escherichia coli or Pseudomonas strains (40–42), for which, however, the mass transfer rate of DBT from the oil to the aqueous phase is a major limiting factor that necessitates the use of cosolvents for higher efficiency (43, 44). In this regard, *Rhodococcus* biocatalysts constitute ideal candidates for genetic enhancement. However, this approach has not been favorable, especially in terms of targeted genetic modifications, owing to their prohibitively low homologous-recombination efficiencies (45, 46). To date, only a few studies have generated desulfurizing *Rhodococcus* strains harboring plasmid-based modifications, which, however, are less preferred for industrial-scale applications due to a lower degree of genetic stability (39, 47–50). To our knowledge, no other studies have reported targeted, genome-based manipulations in IGTS8 or in any other desulfurizing *Rhodococcus* strain.

In the present work, we present the generation of recombinant IGTS8 biocatalysts to investigate the effects of potential gene targets on biodesulfurization activity. More specifically, we implemented a precise, two-step double-crossover genetic engineering approach for the deletion of two sulfur metabolism-related genes, designated *cbs* and *metB*, of *R. qingshengii* IGTS8 (51). Moreover, we provide sequence analyses of the related protein products (CβS and C-γS/L), with emphasis on highly conserved residues of the catalytic core. We present evidence that deletion of the *cbs* gene leads to derepression of biodesulfurization activity mostly for cells grown in the presence of sulfate, whereas biodesulfurization of the Δ*metΒ* engineered strain is more prominent for methionine-grown cells. Furthermore, we report the regulatory role of both CβS and MetB (C-γS/L) in *dszABC* transcription levels in response to the presence of sulfate and methionine, but not cysteine. Thus, we managed to indirectly mitigate the effect of sulfur source repression through targeted genome editing without modifying the native *dsz* operon.

## RESULTS

### Sequence analysis of the *cbs*-*metB* genetic locus.

**FIG 2:**
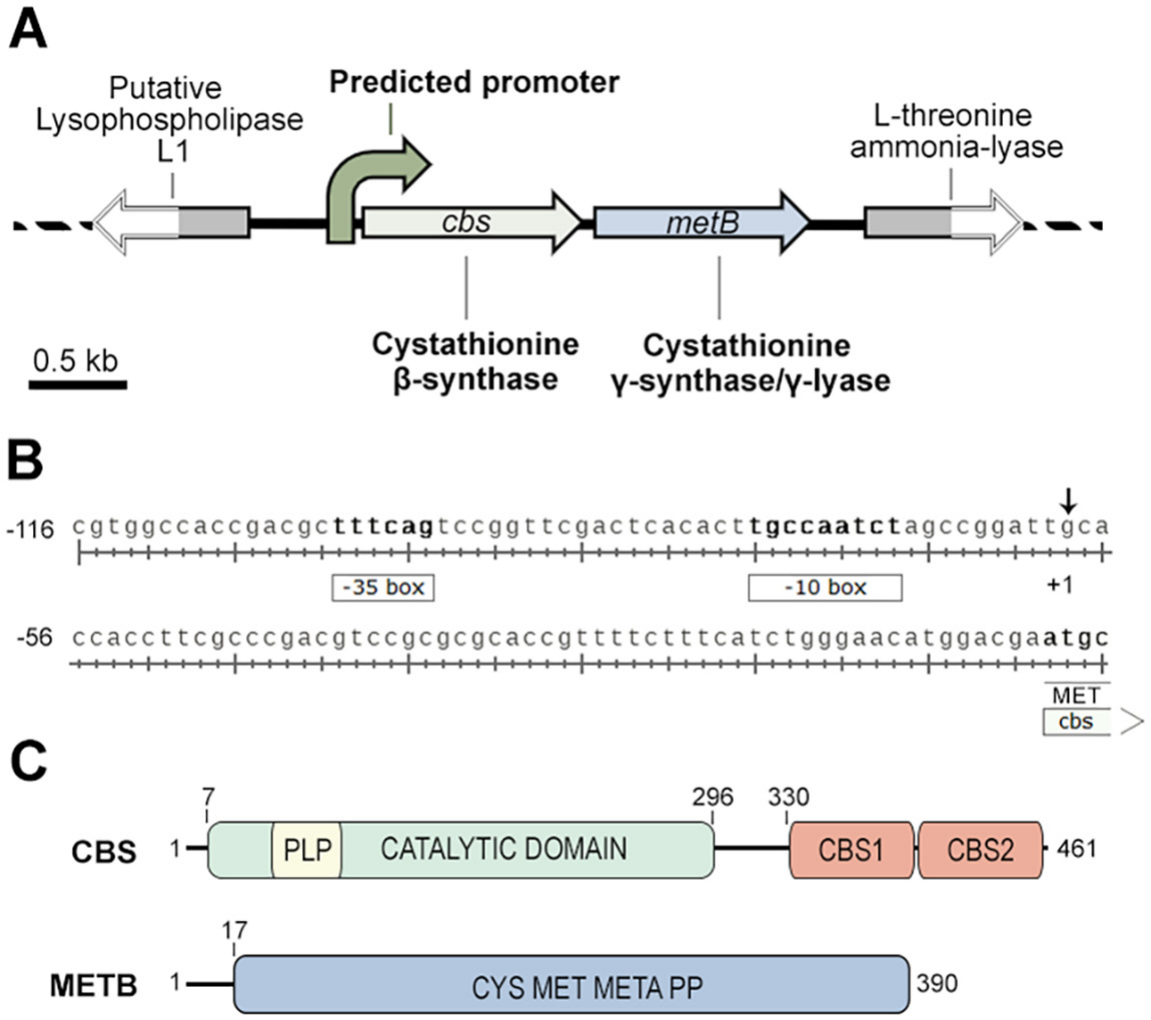
Properties of *cbs-metB* genetic loci and proteins. (A) Scheme of the *cbs-metB* gene cluster. (B) Bacterial promoter predicted sequence. −35 and −10 boxes are displayed, and the arrow indicates the predicted transcription initiation site (+1). (C) Schematic diagram of CβS and MetB (C-γS/L) domain distribution. Cys Met Meta PP, cysteine/methionine metabolism-related PLP-binding domain. See the text for details.

Whole-genome sequencing of *R. qingshengii* IGTS8 (51) revealed a 1,386-bp ORF for *cbs* and a 1,173-bp ORF for *metB*, predicted to encode a CβS and a C-γS/L, respectively. The locus exhibits organization similar to that of strain KA2-5-1 (39) (Fig. 2A). The gene located upstream of the *cbs-metB* locus exhibited 61% identity with M. tuberculosis Rv1075c, a GDSL-like esterase (52), while the gene downstream of *metB* was predicted to encode an l-threonine ammonia-lyase. Analysis of the upstream flanking sequence of *cbs* suggested the presence of a bacterial promoter located ~100 bp before the *cbs* start codon (Fig. 2B).

Based on sequence homology, IGTS8 CβS consists of one N-terminal catalytic domain with the ability to bind PLP (amino acid residues 7 to 296; pfam00291) and two C-terminal CBS regulatory motifs (CBS1, positions330 to 397, and CBS2, positions 403 to 459; pfam00571), commonly referred to as the Bateman module (53, 54). In humans and higher eukaryotes, the protein also harbors an N-terminal heme-binding domain of approximately 70 amino acid residues preceding the catalytic core domain, which has not been found in lower eukaryotes and prokaryotes (36, 55–58). MetB (C-γS/L) consists of a large cysteine/methionine metabolism-related PLP-binding domain (pfam01053), spanning almost the entire protein length (amino acid residues 17 to 390) (Fig. 2C).

**FIG 3:**
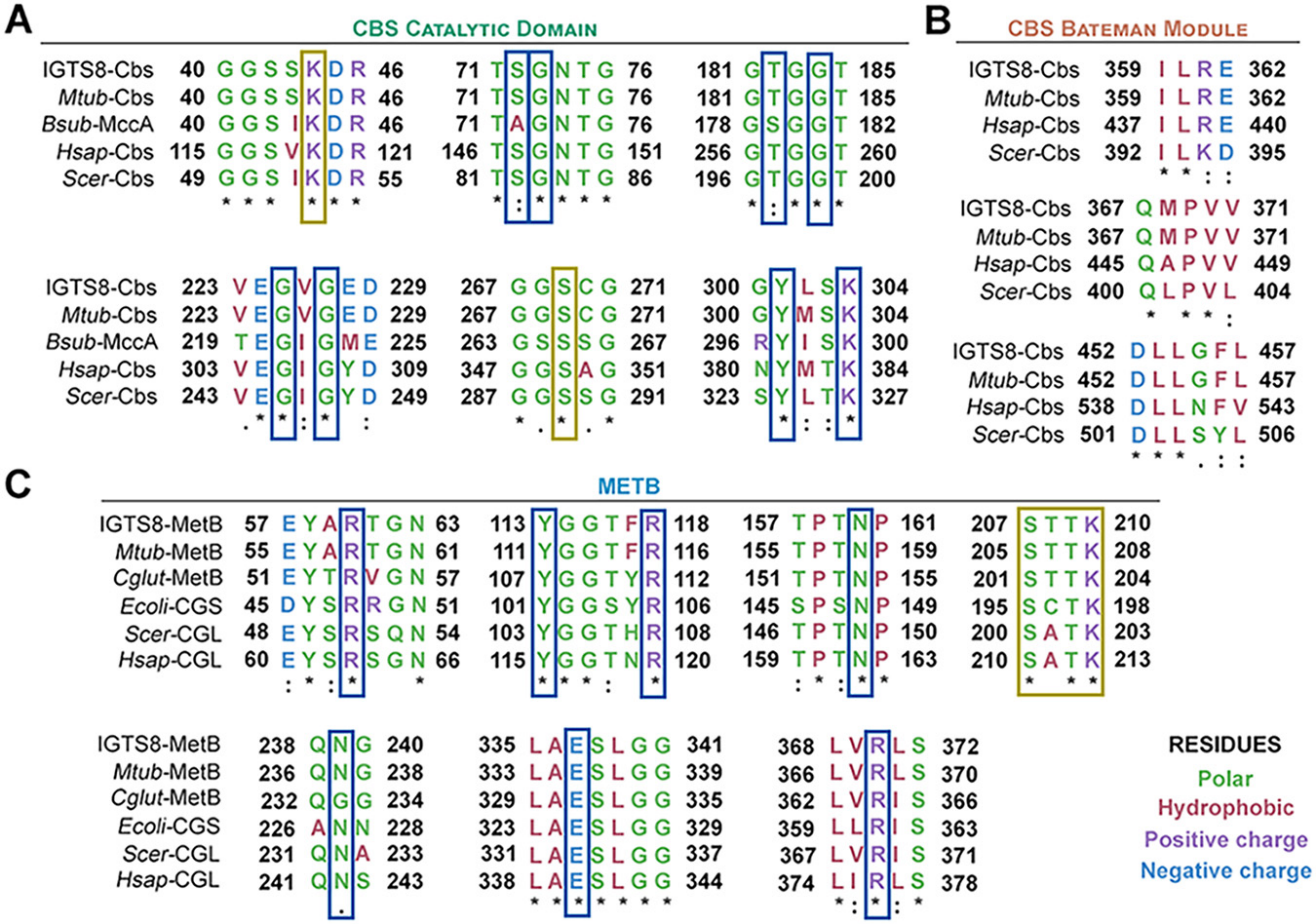
Multiple-sequence alignments of CβS and C-γS/L, displaying only conserved residues configuring the active sites. (A) Comparison of *R. qingshengii* IGTS8 CβS with M. tuberculosis cystathionine β-synthase (UniProt accession no. P9WP51), B. subtilis MccA (UniProt accession no. O05393), human CβS (UniProt accession no. P35520-1), and S. cerevisiae CβS (UniProt accession no. P32582). (B) Comparison of *R. qingshengii* IGTS8 MetB with M. tuberculosis C-γS/L (UniProt accession no. P9WGB7), C. glutamicum CγS (UniProt accession no. Q79VD9), E. coli cystathionine γ-synthase (UniProt accession no. P00935), S. cerevisiae cystathionine γ-lyase (UniProt accession no. P31373), and human cystathionine γ-lyase (UniProt accession no. P32929). All multiple-sequence alignments were done using ClustalO. Asterisks indicate fully conserved residues, colons denote strongly conserved residues, and dots show weakly conserved residues. Residues in yellow boxes participate in PLP binding. Blue boxes indicate residues involved in substrate binding (22, 57).

The translated amino acid sequences of IGTS8 CβS and MetB were compared to those of other known CβS and C-γS/L proteins, respectively. Multiple-sequence alignments revealed the presence of six conserved blocks in the catalytic core of CβS and three in the C-terminal Bateman module of the protein, whereas seven blocks were identified in MetB (Fig. 3). M. tuberculosis CβS showed the highest similarity score to IGTS8 CβS and shared extensive homology across the entire length of the protein (99% coverage, 83% identity). Among the other known CβS homologs, MccA from Bacillus subtilis, an *O*-acetylserine-dependent CβS, showed a 41% overall identity for the compared region (65% coverage), although this protein completely lacked the C-terminal CBS1 and CBS2 regulatory domains. The Homo sapiens and Saccharomyces cerevisiae counterparts showed 40% and 34% similarity, respectively, throughout both the catalytic domain and the Bateman module of the CβS protein. Residues of the catalytic cavity that interact with CβS substrates and the cofactor PLP, were extremely well conserved across the compared sequences (Fig. 3A, blue and yellow boxes, respectively), whereas alignment of the C-terminal CβS regions revealed several highly conserved residues, distributed in three blocks (Fig. 3B).

The MetB (C-γS/L) multiple-sequence alignment included the M. tuberculosis and Corynebacterium glutamicum MetB, the cystathionine γ-synthase from E. coli, and the cystathionine γ-lyases from yeast and human (Fig. 3C). M. tuberculosis and C. glutamicum, which are closely related to *R. qingshengii* IGTS8, possessed homologs with the highest identity scores (73% and 65%, respectively), whereas coverage was high in all MetB sequence alignments (95 to 99%). Notably, the CγS from the Gram-negative bacterium E. coli appeared to have a lower similarity (42%) than the eukaryotic CγLs from S. cerevisiae and H. sapiens (49% and 47%, respectively). This observation is in line with the predicted bifunctionality of IGTS8 MetB as both CγL and CγS, a unique feature that allows the synthesis of l-cysteine through l-methionine via the reverse transsulfuration pathway (25, 59, 60).

Importantly, a nucleotide BLAST search within the IGTS8 genome did not reveal any additional CβS and MetB homologues. In the case of CβS, a tBLASTn search revealed candidates with identities from 33% to 46% (IGTS8_peg5353/CysK1, IGTS8_peg6007/CysK, and IGTS8_peg2567/ThrC), but with low coverage. Concerning IGTS8 MetB, tBLASTn search revealed four putative paralogues (IGTS8_peg1115/CTH, IGTS8_peg771/MetY, IGTS8_peg3888/MetY, and IGTS8_peg5771/MetZ), again with relatively low amino acid identity (34% to 38%). Moreover, compared to M. tuberculosis MetB, these paralogues exhibit 35% to 36% identity and were predicted to be responsible for unrelated functions, except for IGTS8_peg1115 cystathionine gamma-lyase (CTH), which is proposed to also act as a putative cystathionine gamma-lyase (4).

### Effect of C source type on desulfurization activity of IGTS8.

**FIG 4:**
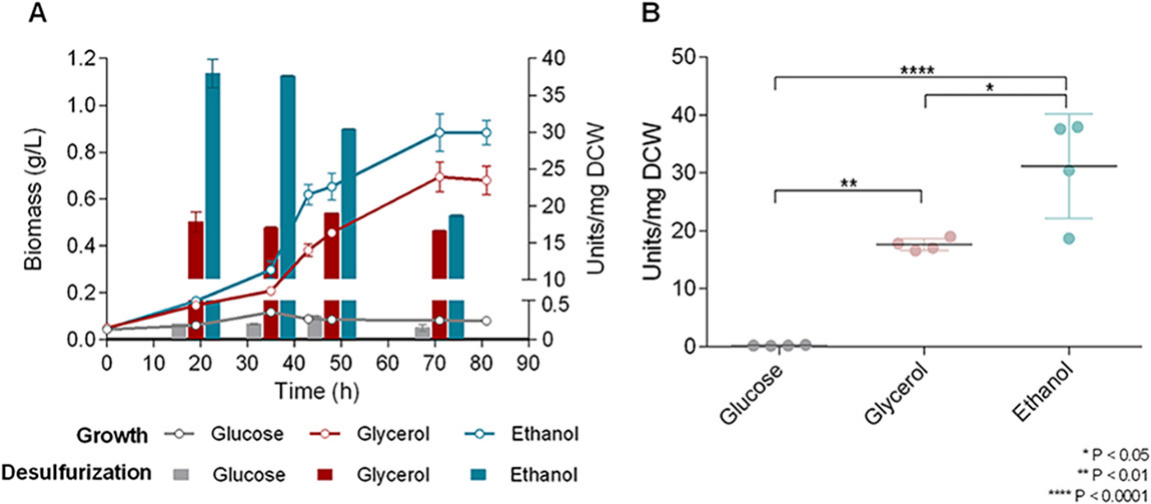
(A) Effect of different carbon sources (0.055 M glucose, 0.110 M glycerol, and 0.165 M ethanol) on growth (biomass) and biodesulfurization activity (units per milligram of cells [dry weight] [DCW]) of *R. qingshengii* IGTS8. DMSO at a concentration of 1 mM was used as the sole sulfur source. (B) Statistical analysis of biodesulfurization results shown in panel A. One-way ANOVA with Tukey’s multiple-comparison test was performed (for more details, see Materials and Methods).

To assess the effect of supplementation with different carbon sources on biodesulfurization capability and to determine the preferred carbon source for *R. qingshengii* IGTS8, we collected samples from actively growing cultures at three different time points (early log, mid-log, and late log phase). Wild-type (wt) *Rhodococcus* cells were grown on either glucose, glycerol, or ethanol as the sole carbon source with 1 mM DMSO as the sole sulfur source (Fig. 4). The highest desulfurization activity for strain IGTS8 was obtained with the use of ethanol as a carbon source. In contrast, utilization of glucose as a carbon source did not lead to a significant increase in attained biomass (0.12 ± 0.02 g/L) or to efficient biodesulfurization (0.30 ± 0.01 U/mg of cells [dry weight]). In fact, cells did not exhibit clear exponential growth even after 80 h of incubation (growth rate [μ_max_] and maximum biomass concentration [*C*_max_] could not be determined). The presence of glycerol as the sole carbon source led to a μ_max_ of 0.075 ± 0.01 h^−1^, a *C*_max_ of 0.80 ± 0.06 g/L, and a biodesulfurization maximum of 19.00 ± 0.04 U/mg of cells [dry weight]. Comparison to the determined μ_max_ (0.081 ± 0.01 h^−1^), *C*_max_ (0.95 ± 0.6 g/L), and measured catalytic activity (38.0 ± 1.9 U/mg of cells [dry weight]) of the same strain upon ethanol supplementation (Fig. 4A) revealed a statistically significant difference for the compared biodesulfurization activities (Fig. 4B) but not for the calculated growth kinetic parameters.

### Growth of knockout mutants on DBT.

To study the role of CβS and C-γS/L (MetB) in the regulation of *dsz* operon expression according to sulfur availability, scarless deletions of the corresponding genes (*cbs* [IGTS8_peg3012] and *metB* [IGTS8_peg3011]) were performed with the use of the pK18mobsacB vector system (see Fig. S1 in the supplemental material; for more details, refer to Materials and Methods). Gene deletions were verified with PCR and DNA sequencing of the PCR products (Text S1). The isogenic Δ*cbs* and Δ*metB* knockout strains retained their ability to grow and desulfurize on liquid minimal media in the presence of 0.1 mM DBT, without the addition of cysteine or methionine (Fig. S2). Ethanol was used as the sole carbon source (0.33 M carbon). The calculated growth rate (μ_max_) of Δ*metB* strain was marginally but not significantly higher than that of wt and Δ*cbs* strains, whereas neither the differences between maximum calculated biomass concentrations (*C*_max_) nor those between the produced 2-hydroxybiphenyl (2-HBP) levels (in micromolar units) were significantly different among the three strains (Fig. S2).

10.1128/mbio.00754-22.1 FIG S1 Schematic representation of knockout strain construction. The constructed vector pK18mobsacB-5′_TG_-3′_TG_ harboring the upstream (5′_TG_) and downstream (3′_TG_) flanking regions of the target gene (TG) is inserted to the bacterial cell of the recipient strain (wt *R. qingshengii* IGTS8) via conjugal transfer. In the first step of the gene deletion process, homologous recombination takes place between the transferred DNA (vector) and the chromosomal DNA of the recipient strain, resulting in either 5′ (upstream) or 3′ (downstream) plasmid incorporation. The generated transconjugants are kanamycin resistant (Kan^r^) and sucrose sensitive (Suc^s^), allowing kanamycin resistance selection. As an additional control, transconjugants should exhibit no growth in the presence of 10% sucrose. The validated upstream or downstream merodiploids are grown in the absence of selective pressure (without antibiotic addition) to induce the second homologous recombination event. Depending on the flanking regions involved (5′ or 3′), the result is either a knockout of the target gene (*tg*Δ) or a reversion to the wt, while the rest of the integrated vector is excised from the genome. The generated strains are sucrose resistant; thus, any incomplete excision events are removed in the final step of the process, with selection in the presence of 10% sucrose. Additionally, kanamycin sensitivity is restored at this point. Single colonies are isolated, and knockouts are confirmed with PCR using the primer pair 5F-check and 3R-check. The PCR products are further verified with DNA sequencing. Download FIG S1, TIF file, 0.4 MB. Copyright © 2022 Martzoukou et al. 2022 Martzoukou et al. https://creativecommons.org/licenses/by/4.0/ This content is distributed under the terms of the Creative Commons Attribution 4.0 International license.

10.1128/mbio.00754-22.2 FIG S2 Growth and 2-HBP production in the presence of DBT as the sole sulfur source. Growth (biomass) and 2-HBP concentration in the culture medium of wt, Δ*cbs*, and Δ*metB* strains in the presence of 0.1 mM DBT as the sole sulfur source. Ethanol (0.165 M) was the sole carbon source. For measurements of produced 2-HBP (in micromolar units) at 0, 24, 48, and 72 h, an aliquot of whole culture was harvested in Eppendorf tubes and an equal volume of acetonitrile was added. Suspensions were vortexed vigorously, centrifuged (14.000 × *g*; 10 min), and 2-HBP concentration was determined in the collected supernatant through HPLC. Calculated μ_max_ and *C*_max_ values are included in the upper left inset (*n* = 3). Download FIG S2, TIF file, 0.2 MB. Copyright © 2022 Martzoukou et al. 2022 Martzoukou et al. https://creativecommons.org/licenses/by/4.0/ This content is distributed under the terms of the Creative Commons Attribution 4.0 International license.

10.1128/mbio.00754-22.8 TEXT S1 DNA sequences of *cbs-metB* genetic loci for wild-type and recombinant strains. Download Text S1, PDF file, 0.1 MB. Copyright © 2022 Martzoukou et al. 2022 Martzoukou et al. https://creativecommons.org/licenses/by/4.0/ This content is distributed under the terms of the Creative Commons Attribution 4.0 International license.

### Recombinant strains exhibit increased desulfurization activity when grown on repressive sulfur sources.

**FIG 5:**
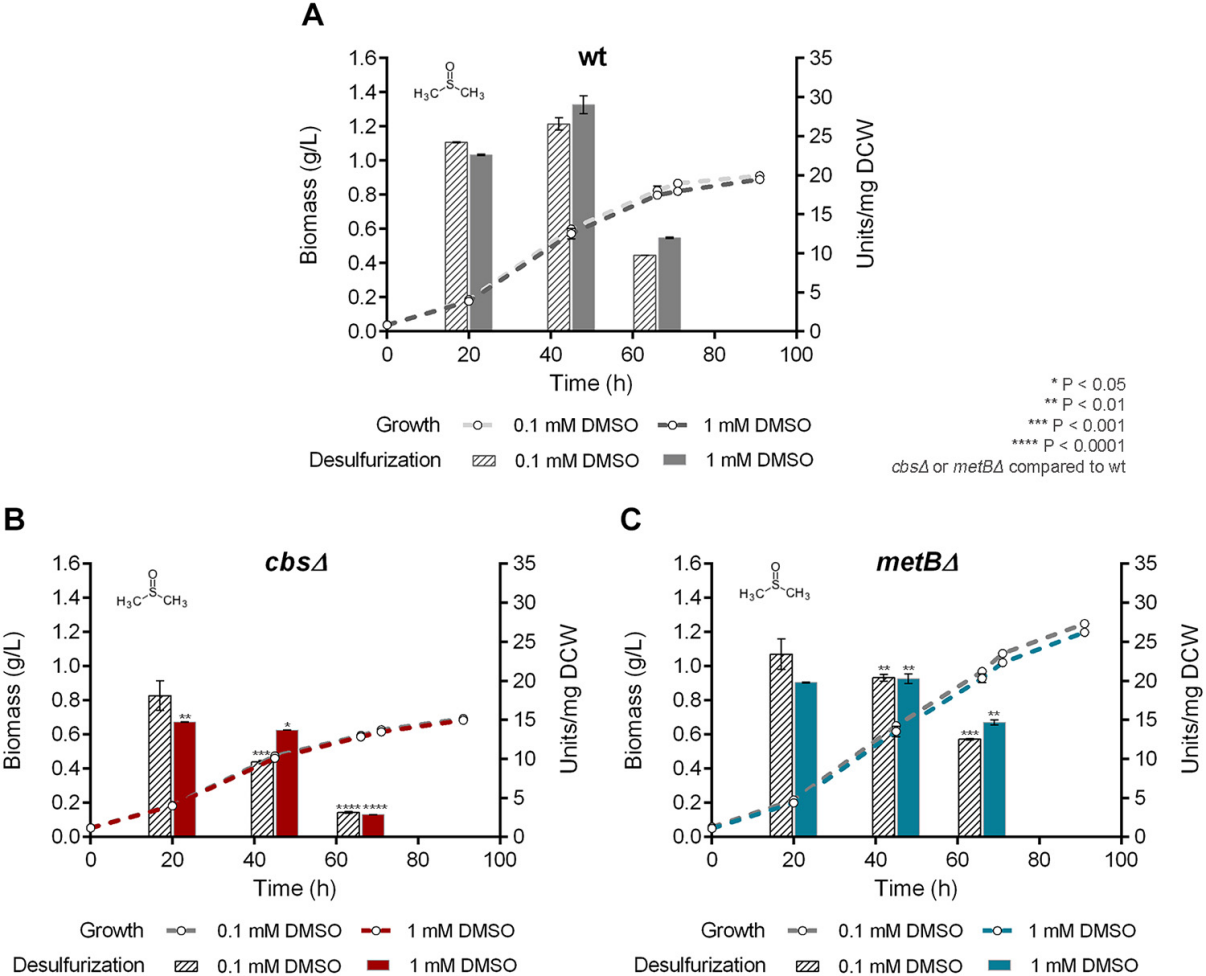
Effect of DMSO as the sole sulfur source on growth (biomass) and biodesulfurization activity (units per milligram of cells [dry weight] [DCW]) of wt (A), Δ*cbs* (B), and Δ*metB* (C) strains grown on CDM in the presence of low (0.1 mM) and high (1 mM) DMSO concentrations. Ethanol (0.165 M) was the sole carbon source in the culture medium.

**FIG 6:**
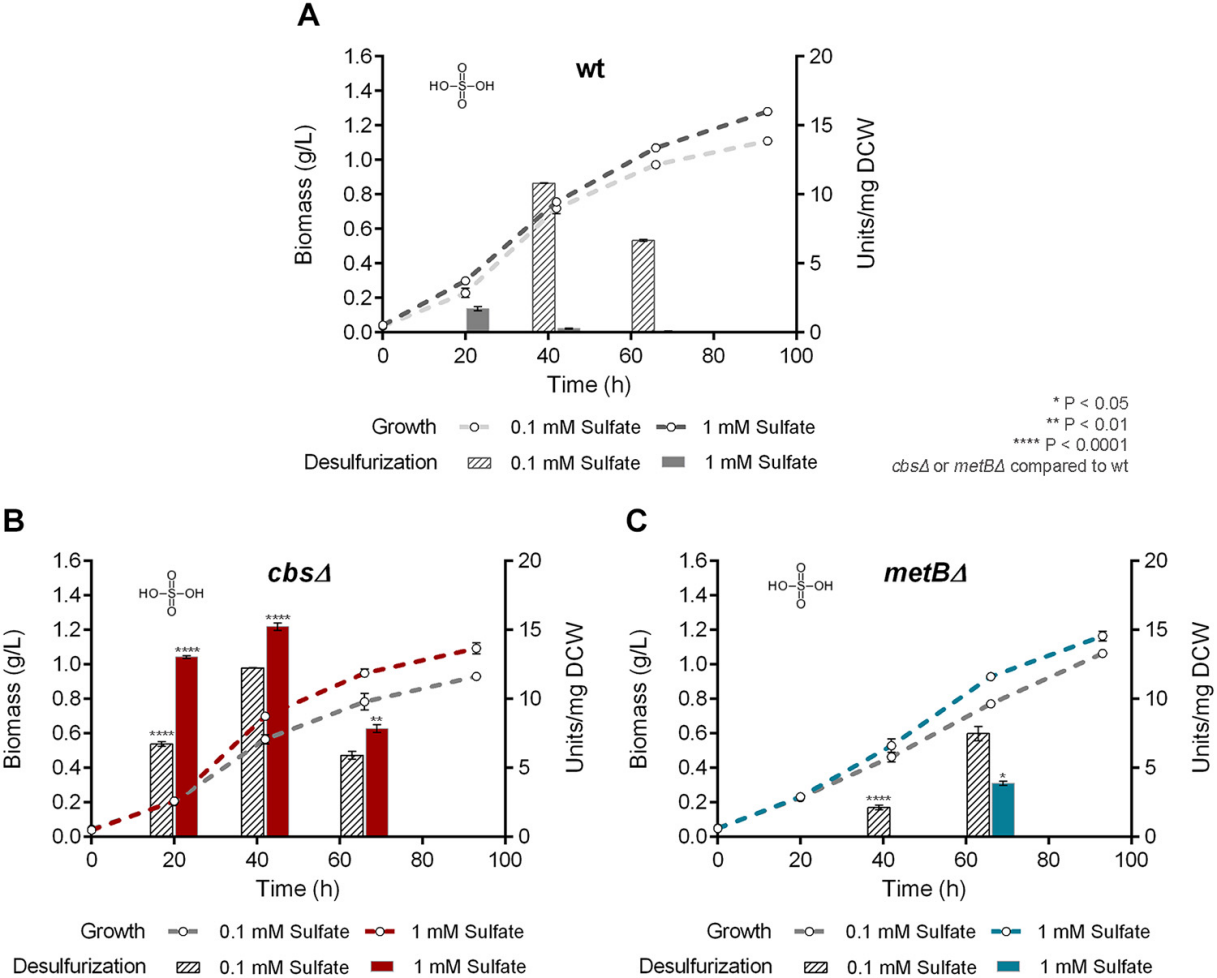
Effect of sulfate as the sole sulfur source on growth (Biomass; g/L) and biodesulfurization activity (units/mg of cells [dry weight] [DCW]) of wt (A), Δ*cbs* (B), and Δ*metB* (C) strains grown on CDM in the presence of low (0.1 mM) and high (1 mM) sulfate concentrations. Ethanol (0.165 M) was the sole carbon source in the culture medium.

**FIG 7:**
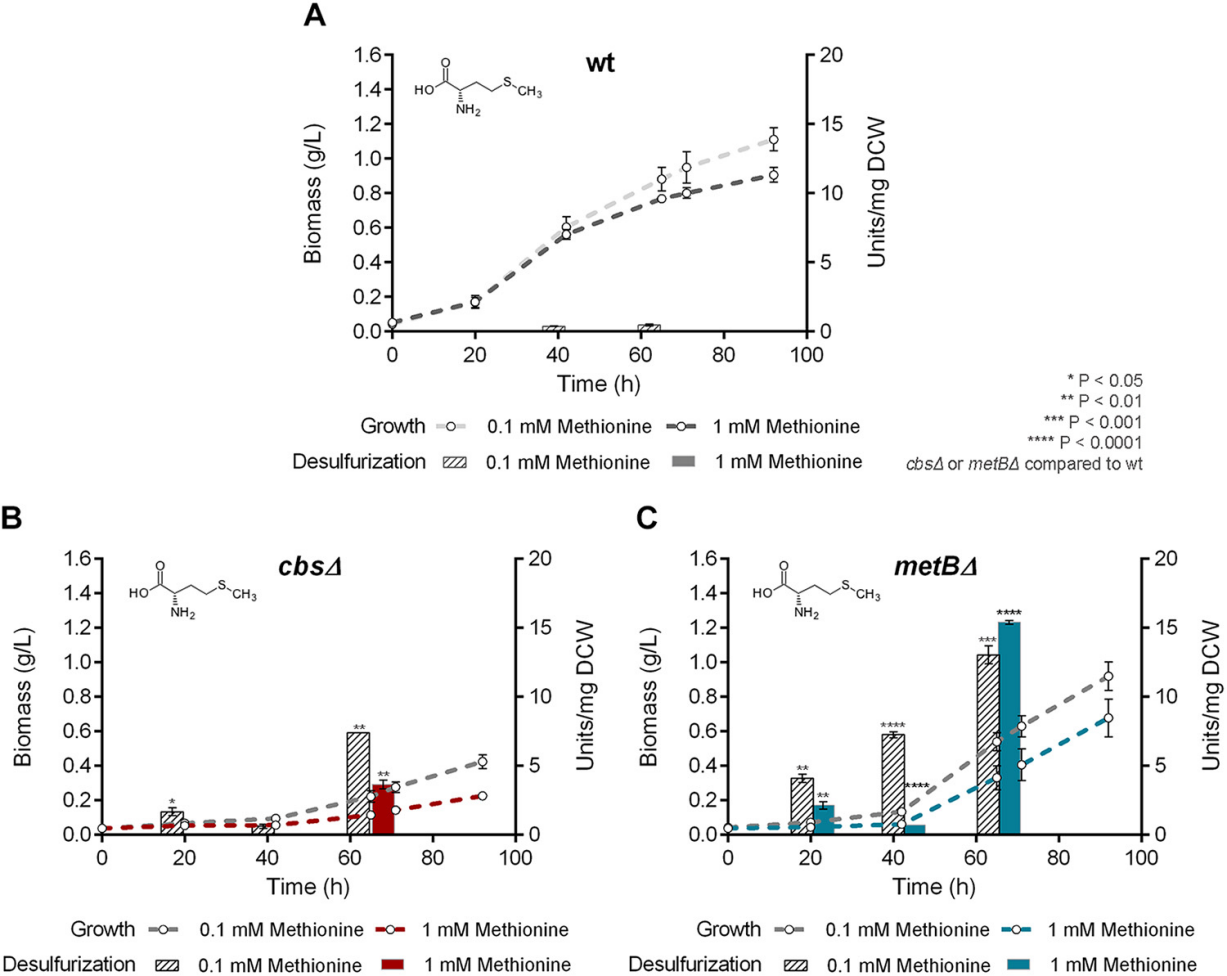
Effect of methionine as the sole sulfur source on growth (biomass) and biodesulfurization activity (units per milligram of cells [dry weight] [DCW]) of wt (A), Δ*cbs* (B), and Δ*metB* (C) strains grown on CDM in the presence of low (0.1 mM) and high (1 mM) methionine concentrations. Ethanol (0.165 M) was the sole carbon source in the culture medium.

**FIG 8:**
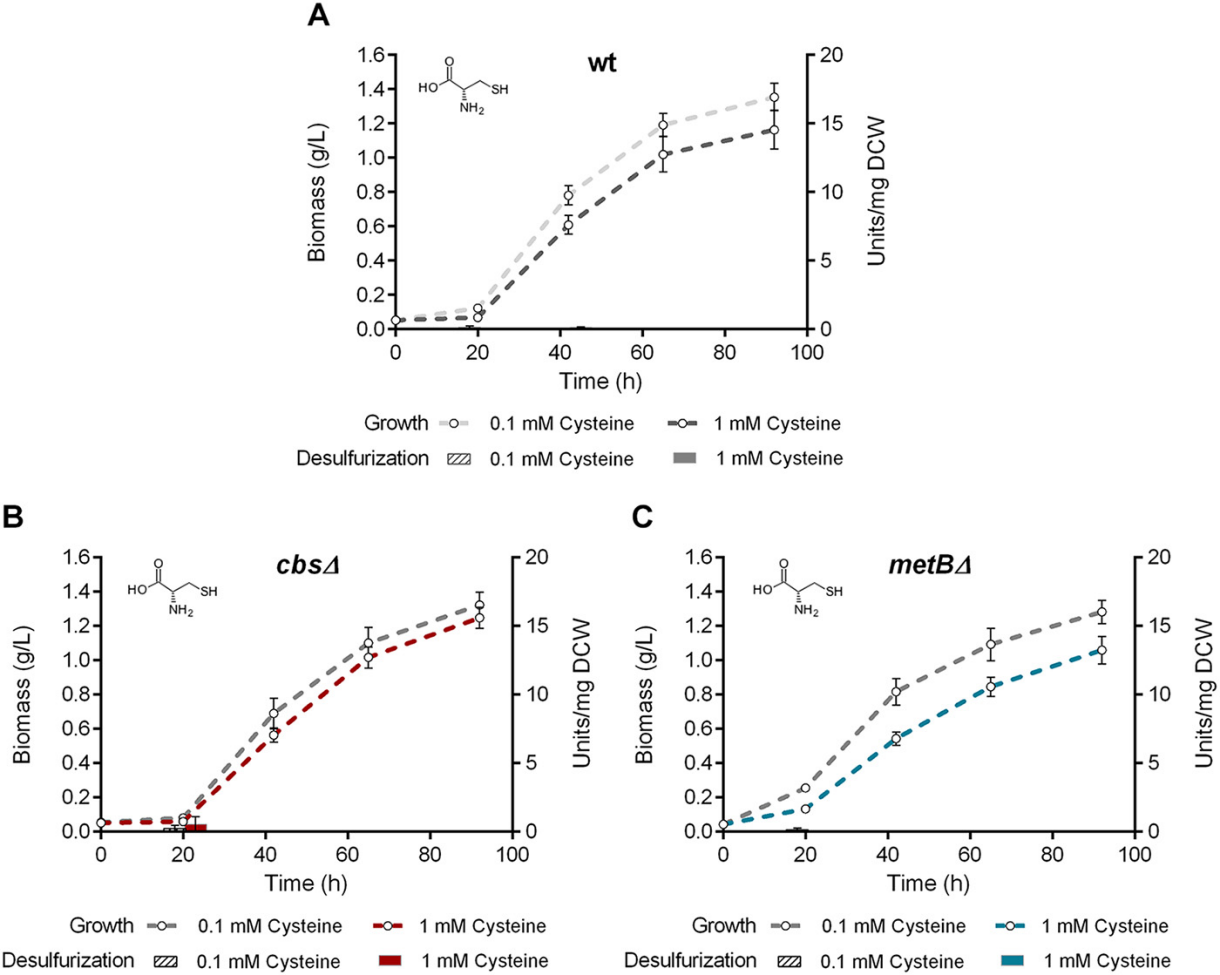
Effect of cysteine as the sole sulfur source on growth (biomass) and biodesulfurization activity (units per milligram of cells [dry weight] [DCW]) of wt (A), Δ*cbs* (B), and Δ*metB* (C) strains grown on CDM in the presence of low (0.1 mM) and high (1 mM) cysteine concentrations. Ethanol (0.165 M) was the sole carbon source in the culture medium.

We investigated the effect of *cbs* and *metB* deletions on growth and desulfurization capability of *R. qingshengii* IGTS8, by comparing the isogenic Δ*cbs*, Δ*metB*, and wt strains. Interestingly, growth phase-dependent variations of biodesulfurization were observed for all strains that retained the ability to desulfurize under the influence of different medium compositions, whereas statistical analysis revealed that growth kinetic parameters of each strain were not significantly affected by the amount of sulfur source added (Fig. 5 to 8 and Table S2). The biodesulfurization activity of resting cells was first determined for cultures grown under nonrepressive conditions, with the supplementation of DMSO (Fig. 5). Comparison of *C*_max_ values between the wt and each of the two recombinant strains showed statistically significant differences for each concentration of DMSO supplement (*P* < 0.05), whereas differences between the respective μ_max_ values were nonsignificant. Comparison of biodesulfurization activities between different strains grown on DMSO showed a statistically significant adverse effect of CβS depletion, whereas the Δ*metB* strain was affected primarily during mid-log and late log phases (*P* < 0.05) (see Fig. 5B and C for more details). Additionally, nonsignificant concentration-dependent (0.1 versus 1 mM DMSO) variations of biodesulfurization activities were observed for each strain studied.

10.1128/mbio.00754-22.6 TABLE S2 Growth kinetic parameters (μ_max_ and *C*^max^_*X*_) obtained by fitting of biomass concentration versus time experimental values to the logistic equation. Download Table S2, PDF file, 0.2 MB. Copyright © 2022 Martzoukou et al. 2022 Martzoukou et al. https://creativecommons.org/licenses/by/4.0/ This content is distributed under the terms of the Creative Commons Attribution 4.0 International license.

To determine the effect of *cbs* and *metB* gene deletions under biodesulfurization-repressive conditions, we performed growth studies with the supplementation of sulfate, methionine, or cysteine as the sole sulfur source (Fig. 6 to 8, respectively, and Table S2) followed by resting cells’ desulfurization assays. Sulfate addition in the bacterial culture efficiently repressed the desulfurization activity of the wt strain, only when a high concentration was used (1 mM; *P* < 0.0001) (Fig. 6A). The specific growth rate of the Δ*metB* but not the Δ*cbs* strain exhibited a significant reduction in the presence of 0.1 mM sulfate (*P* < 0.01 compared to the wt). Values of calculated growth kinetic parameters are reported in Table S2. Deletion of *cbs* and *metB* led to nonsignificant variations of *C*_max_ for both sulfate concentrations, compared to the wt. Notably, desulfurization was enhanced 9-fold for Δ*cbs* in the presence of 1 mM sulfate, reaching up to 15.23 ± 0.27 U/mg of cells [dry weight] at mid-log phase compared to the wt (*P* < 0.0001) (Fig. 6A and B). The MetB-depleted strain exhibited significant biodesulfurization activity only during the late exponential phase for 1 mM sulfate (*P* < 0.05; 7.49 ± 0.51 U/mg of cells [dry weight]) (Fig. 6C). Moreover, concentration-dependent variations in biodesulfurization activities were validated for wt (*P* < 0.0001, 45 h and 65 h), Δ*cbs* (*P* < 0.0001, 20 h; *P* < 0.01, 45h), and Δ*metB* (*P* < 0.05, 45 h; *P* < 0.001, 65 h) strains, grown in the presence of sulfate (0.1 versus 1 mM).

An unexpected finding is that the absence of CβS and MetB seemed to have a negative effect on methionine-based growth (Fig. 7 and Table S2). Recombinant Δ*metB* exhibited lower μ_max_ values than the wt strain (*P* < 0.05, 0.1 mM; *P* < 0.0001, 1 mM), whereas the calculated *C*_max_ for growth on 1 mM methionine appeared to be significantly increased (*P* < 0.001). Importantly, growth kinetic parameters could not be determined for the Δ*cbs* strain, due to poor growth. Concerning biodesulfurization activities of strains grown on methionine, the wt was completely unable to desulfurize DBT even in the presence of a low concentration (0.1 mM; 0.46 ± 0.06 U/mg of cells [dry weight]) (Fig. 7A). The biodesulfurization activity of Δ*cbs* strain became more evident for cells harvested after 65 h of growth on both methionine concentrations (7.34 ± 0.05 U/mg of cells [dry weight], 0.1 mM, and 3.65 ± 0.31 U/mg of cells [dry weight], 1 mM; *P* < 0.01 compared to wt) (Fig. 7B). In contrast, the Δ*metB* strain exhibited remarkable desulfurization activity after 65 h of growth on both low and high concentrations of the sulfur source (13.1 ± 0.65 U/mg of cells [dry weight], *P* < 0.001, and 15.4 ± 0.13 U/mg of cells [dry weight], *P* < 0.0001, respectively) (Fig. 7C). Concentration-dependent variations (0.1 versus 1 mM methionine) were also observed in a comparison of biodesulfurization activities for the recombinant Δ*cbs* strain (*P* < 0.01, 20 h; *P* < 0.0001, 65 h) and Δ*metB* strain (*P* < 0.001, 20 h; *P* < 0.0001, 45 h and 65 h) and *C*_max_ for the Δ*metB* strain (*P* < 0.05).

Importantly, certain biodesulfurization activity variations were observed for recombinant cells grown on different sulfate or methionine concentrations but not for DMSO or cysteine. For example, the Δ*cbs* strain showed a preference for a higher sulfate and a lower methionine content, whereas the Δ*metB* strain preferred the higher methionine concentration only for late-log-phase-harvested cells. Concerning growth phase-dependent variations of biodesulfurization activity, in most cases, maximum values were attained mid-log and minimum biodesulfurization activity was observed for late-exponential-phase cultures. Exceptions were the Δ*metB* strain supplemented with sulfate and both recombinant strains grown on methionine, as they exhibited maximum biodesulfurization after prolonged growth (65 h).

Cysteine supplementation as the sole sulfur source in the culture medium resulted in complete inability of all strains (wt, Δ*cbs* and Δ*metB*) to desulfurize DBT, even in the presence of low sulfur content (Fig. 8). However, growth was efficient in all cases, as maximum biomass concentrations reached 1.37 ± 0.065 g/L (Table S2). Differences between the μ_max_ or *C*_max_ values of recombinant strains compared to the respective wt values, as well as concentration-dependent variations within strains (0.1 versus 1 mM cysteine), were nonsignificant. We also tested the effect of cysteine supplementation at 10 mM for all strains, as cysteine is known to be toxic at high concentrations (61). Calculated *C*_max_ values of the wt and Δ*cbs* strains, but not the Δ*metB* strain, were significantly reduced in the presence of high exogenous cysteine concentration (10 mM), compared to lower cysteine concentrations (wt, 0.1 mM versus 10 mM, *P* < 0.05; Δ*cbs*, 0.1 or 1 mM versus 10 mM, *P* < 0.0001) (Fig. S3). Growth rates of all strains were not significantly affected by the higher cysteine concentration.

10.1128/mbio.00754-22.3 FIG S3 Effect of cysteine supplementation at a high concentration. Growth curves (biomass) and desulfurization activities (units of 2-HBP per milligram of cells [dry weight]) of wt, Δ*cbs*, and Δ*metB* strains in the presence of 10 mM cysteine as the sole sulfur source. Ethanol (0.165 M) was the sole carbon source in the culture medium. Calculated μ_max_ and *C*_max_ values are included in the upper left inset. Download FIG S3, TIF file, 0.2 MB. Copyright © 2022 Martzoukou et al. 2022 Martzoukou et al. https://creativecommons.org/licenses/by/4.0/ This content is distributed under the terms of the Creative Commons Attribution 4.0 International license.

### Deletion of *cbs* or *metB* leads to increased transcriptional levels of *dszABC* desulfurization genes in the presence of selected S sources.

**FIG 9:**
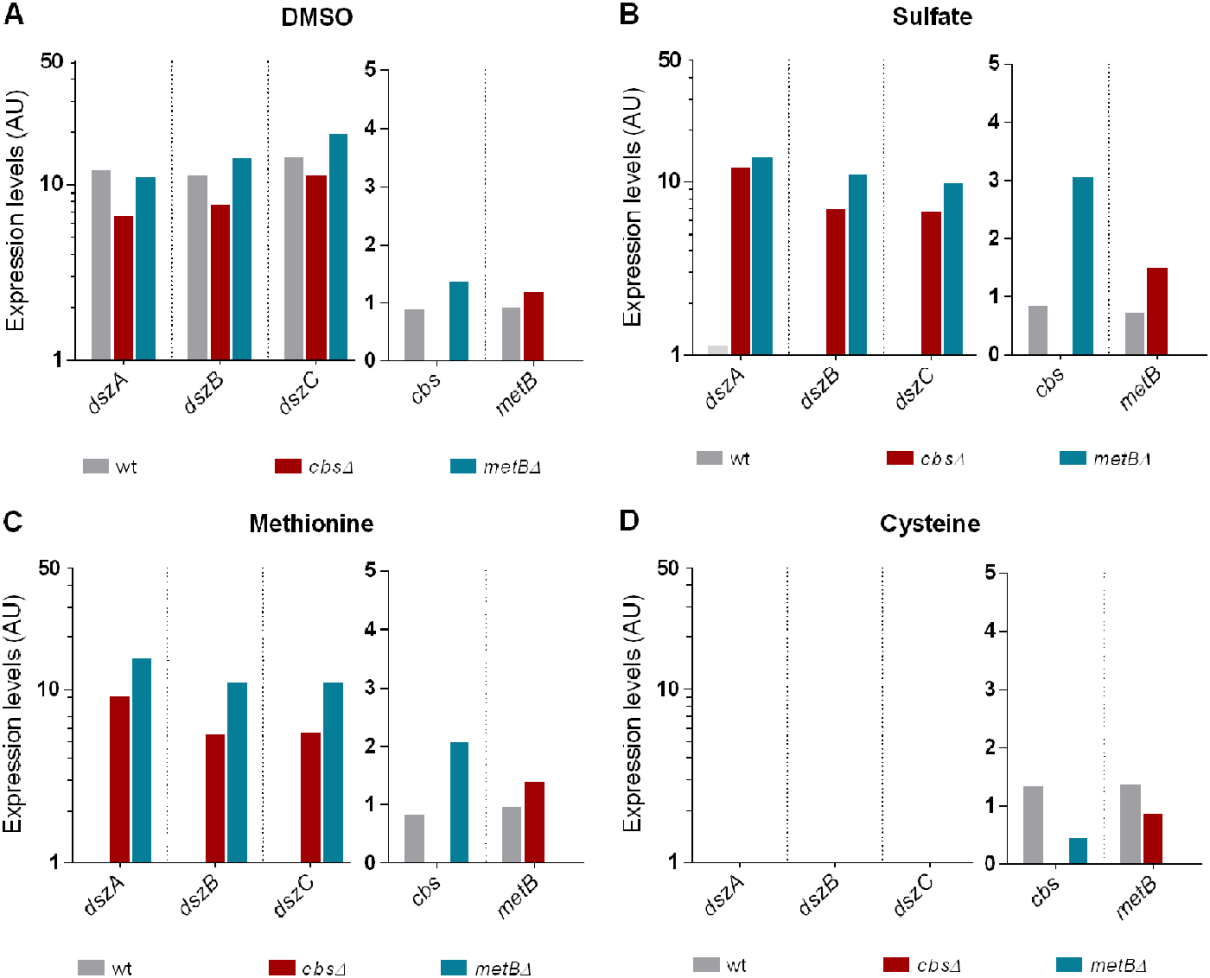
Comparison of *dszA*, *dszB*, *dszC*, *cbs*, and *metB* transcriptional levels for the wt and the Δ*cbs* and Δ*metB* isogenic strains in the presence of 1 mM (A) DMSO, (B) sulfate, (C) methionine, or (D) cysteine. Samples were collected from mid-log-phase cultures, and expression levels relative to the calibrator sample are reported. A logarithmic scale was used for *dszABC*. For details, see Materials and Methods. Ethanol (0.165 M) was the sole carbon source in the culture medium. AU, arbitrary units.

To elucidate the effect of *cbs* and *metB* deletions on the transcriptional levels of *dszABC* desulfurization genes, as well as the regulation of *dsz*, *cbs*, and *metB* gene expression in response to sulfur availability, we performed a series of qPCRs for wt, Δ*cbs* and Δ*metB* strains under repressive and nonrepressive conditions. In the presence of DMSO as the sole sulfur source (Fig. 9A), *dszABC* genes were efficiently expressed regardless of *cbs* or *metB* deletions. Additionally, *cbs* and *metB* transcriptional levels did not exhibit significant changes in the presence of DMSO, compared to wt. Sulfate or methionine supplementation (Fig. 9B and C, respectively) led to repression of *dszABC* operon expression for the wt strain, while both Δ*cbs* and Δ*metB* knockout strains exhibited increased expression levels of the desulfurization genes (Δ*cbs* sulfate, *P* < 0.01; Δ*metB* sulfate, *P* < 0.001; Δ*cbs* methionine, *P* < 0.05; Δ*metB* methionine, *P* < 0.001, compared to wt sulfate or wt methionine, respectively). Moreover, under the same conditions, *metB* and *cbs* gene expression appeared slightly elevated, but not significantly different, for the Δ*cbs* and Δ*metB* strains, respectively, compared to the wt (Fig. 9B and C). Interestingly, loss of *dszABC* transcription was observed in the presence of cysteine (Fig. 9D), not only for the wt but also for the two knockout strains. Furthermore, *cbs* and *metB* expression levels did not exhibit significant changes between different *cbs^+^* or *metB^+^* strains grown on the same sulfur source or for the same strain grown on different sulfur sources (Fig. 9A to C). Overall, the results were in line with the observed sulfate- and methionine-related derepression of the desulfurization activity in response to *cbs* and *metB* deletions.

## DISCUSSION

Certain aspects of sulfur metabolism are well characterized in several Gram-positive bacteria; however, the regulation of sulfur assimilation-related gene expression in rhodococcal desulfurizing species remains unclear. This is a curiously paradoxical situation, given that *R. qingshengii* IGTS8 is the most extensively studied biocatalyst for industrial biodesulfurization applications.

In contrast to the approaches that included the *in silico* modeling of sulfur assimilation (12) and the most recent proteomics and metabolomics analyses in strain IGTS8 (4), in the present work, we performed targeted and precise editing of the *R. qingshengii* IGTS8 genome, generating recombinant biocatalysts that harbor gene deletions of the two enzymes predicted to be involved in the reverse transsulfuration pathway. Importantly, primary amino acid sequence analyses of IGTS8 CβS and C-γS/L (MetB) suggested the presence of highly conserved residue blocks that participate in active-site configuration and binding of substrates and PLP cofactor. The high degree of similarity between the two IGTS8 enzymes and their respective counterparts found in the closely related species (62) M. tuberculosis (83% identity for CβS, 73% identity for MetB) suggested a conserved function for these proteins as cystathionine β-synthase and cystathionine γ-lyase, respectively. This result is in line with previous reports suggesting the existence of an operational reverse transsulfuration pathway for the genus *Rhodococcus* (4, 39). In accordance with our sequence analyses and multiple-alignment results, the sulfur assimilation model proposed by Hirschler et al. (4) identified CβS as a cystathionine β-synthase and MetB as a cystathionine γ-lyase (CγL). Notably, annotation of the IGTS8 genome (4) predicts the existence of a second CγL (IGTS8_peg1115/CTH), which, however, exhibited relatively low amino acid identity to the MetB proteins of M. tuberculosis and IGTS8 (35% and 38%, respectively). Therefore, the possibility for an auxiliary or cryptic role of CTH in cysteine biosynthesis from cystathionine cannot be excluded.

To perform growth and desulfurization assays with the use of a single carbon source, we compared the effects of glucose, glycerol, and ethanol supplements at 0.33 M carbon. Notably, growth of wt IGTS8 was inefficient in the presence of glucose as the sole carbon source, when DMSO was used as the sole sulfur source. Although glucose has been generally used as a carbon source in previous studies, it was either in combination with a different type of sulfur source, or both glucose and DMSO present at elevated concentrations, or used in cosupplementation with glycerol (5, 14, 50, 63, 64). Thus, our findings might point at a limited uptake and/or reduced assimilation of this carbon source in the medium composition we used. To our knowledge, ethanol superiority as a sole carbon source was verified for R. erythropolis KA2-5-1 (65) and for transformants of *R. qingshengii* CW25, but only in comparison to glucose (50), and very recently for *R. jostii* RHA1 in comparison to glucose, *n*-octane, and 1-butanol (14). In the current study, ethanol was validated as the preferred carbon source for desulfurization activity of IGTS8, probably due to the additional reducing power (NADH) that it provides during its catabolism, a fact that has also been predicted through flux-based analysis of sulfur metabolism in R. erythropolis (12) and was thus used as a sole carbon source in experiments where the sulfur source type was the variable.

Bacterial cultures of both the wt and recombinant strains in the presence of DBT as a sole sulfur source showed no significant differences in terms of growth profile and biodesulfurization activity. Considering that IGTS8 restricts sulfur assimilation in the DBT cultures (4), that the native *dsz* locus of the strains was not modified, and that the biodesulfurization phenotype is not repressed by DBT (5), this finding likely indicates that under nonrepressive conditions (biodesulfurization), all strains acquire sulfur from DBT and assimilate it into biomass at comparable levels.

Overall, our findings revealed that biodesulfurization activity is largely affected by the type and concentration of the sulfur source available and under certain conditions by the growth phase of the culture, possibly indicating a temporal shift in Dsz enzyme availability. In the case of sulfate supplementation as the sole sulfur source, sulfide production can occur via a four-step process, with the intermediary formation of sulfite. The availability of sulfate is known to stimulate divergent routes for sulfate/sulfite reduction, while the latter serves as a metabolic branching point (4). In turn, sulfide can either enter the MetZ/MetY-dependent routes for homocysteine production or be converted to cysteine via CysK. According to the sulfur assimilation model proposed recently by Hirschler et al. (4), under sulfate-rich conditions, the reverse transsulfuration metabolic reactions probably serve as the primary route for cysteine biosynthesis from methionine, whereas the CysK-dependent alternative route likely operates as a secondary pathway. According to the same study (4), protein levels of CβS and MetB were slightly higher but not significantly different in the sulfate cultures compared to the DBT cultures. Based on these findings, cysteine biosynthesis in the presence of sulfate as the sole sulfur source might proceed through the CysK-dependent alternative route, upon CβS or MetB depletion, whereas MetZ might lead to the biosynthesis of homocysteine from sulfide. This in turn can be converted to cystathionine in the Δ*metB* (*cbs*^+^) mutant or to methionine via MetH.

The enhanced biodesulfurization activities of recombinant strains, especially the Δ*cbs* strain, were confirmed by the qPCR results in the presence of 1 mM sulfate. A discrepancy was observed between the biodesulfurization activity and the corresponding transcript levels, as *dsz* expression in the Δ*metB* strain appeared to be slightly higher than that of the Δ*cbs* strain, although the maximum biodesulfurization activity of the latter was higher. In this context, alterations in transcript levels of *dszABC* genes (mRNA) may affect the biodesulfurization activity (determined by the measured 2-HBP), but the correlation between the two is not necessarily proportional, since other factors involved in the process could exhibit variations between the different recombinant strains. These factors include *dsz* mRNA stability, relative Dsz protein expression levels, protein stability/turnover, metabolic inhibition, DBT uptake, ethanol catabolism, and hence NADH availability (12, 65, 66). Thus, preferably a combination of the two methods (i.e., transcriptional levels and biodesulfurization assays) can provide clearer insight on the regulation of *dsz-*mediated sulfur assimilation.

When the nonrepressive sulfur source DMSO is used, it can be reduced to dimethyl sulfide (DMS) and subsequently converted to methanethiol, which in turn is oxidized to generate sulfide (67). CysK, the enzyme responsible for the conversion of sulfide to cysteine, was significantly more abundant under sulfate starvation conditions (DBT) (4). Thus, considering also the nonrepressive nature of DMSO, one would expect sulfur assimilation and cysteine biosynthesis to operate normally in the recombinants and the wt strain, possibly involving the CysK-mediated direct sulfhydrylation pathway. However, comparison of biodesulfurization activities for different strains grown in the presence of DMSO showed a negative effect of CβS—and, to a lesser extent, MetB—depletion, even though *dsz* transcriptional levels did not differ significantly between the wt and the mutants. DMSO is used widely as a nonrepressive sulfur source (64, 68). Nevertheless, metabolic alterations associated with reverse transsulfuration enzyme depletion, in combination with DMSO-induced stress, which often associates with cytological alterations and growth inhibition (69–72), may cause this adverse effect on biodesulfurization activities in recombinant strains. The Δ*cbs* and Δ*metB* knockout strains did not require supplemental cysteine or methionine, as evidenced by their ability to grow in the presence of DBT, DMSO, and sulfate as sole sulfur sources. The prototrophic nature of recombinants suggests the existence of alternative operating routes for sulfur-containing amino acid biosynthesis. Cysteine biosynthesis via the CysK-mediated direct sulfhydrylation pathway or other operational cysteine biosynthesis routes could partly compensate for the reduced cysteine supply, in the absence of CβS or MetB, in our growth and biodesulfurization studies. In contrast, the CTH-mediated pathway for cysteine production from cystathionine could explain the nonessentiality only of MetB, not of CβS.

Providing methionine as the sole sulfur source to the Δ*cbs* or the Δ*metB* strain might lead to an overproduction of homocysteine or cystathionine, respectively, given that the reverse transsulfuration pathway would no longer be functional. Following an alternative route, not the typical MetK-dependent route, methionine could be converted to methanethiol and eventually to sulfide, thus increasing the precursor molecules for CysK-mediated cysteine biosynthesis. The enhanced biodesulfurization activity of the Δ*metB* strain, and to a lesser extent that of Δ*cbs* strain, is supported by the elevated *dszABC* transcript levels of both knockouts in the methionine cultures. Moreover, the low growth rate of the Δ*cbs* strain is fully in line with the low growth yield reported for the transposon-disrupted *cbs* strain R. erythropolis KA2-5-1 in the presence of 5 mM methionine (39).

We assume that although CβS and MetB are nonessential, their depletion could lead to diverse effects, including lower cysteine availability, redirection of metabolic precursors toward competing pathways, and/or accumulation of intermediary toxic metabolites that could potentially inhibit growth (61, 73). Therefore, variations in the biodesulfurization activities and growth kinetics of recombinant strains in response to different sulfur concentrations or different sulfur source types may involve alterations in the levels of CβS and MetB substrates, i.e., homocysteine and serine for the Δ*cbs* strain and cystathionine for the Δ*metB* strain. In turn, these could signal the differential regulation of alternative sulfur assimilation routes, such as the MetE/MetH-mediated conversion of homocysteine to methionine, or cysteine production from serine with the intermediary formation of OAS via the CysE-CysK bienzyme complex (74, 75). Changes in pathway fluxes of recombinant strains might affect the biodesulfurization activity at the posttranscriptional level or exert an indirect effect, given that *dsz* expression levels do not seem to differ significantly for the knockout strains in the presence of sulfate or methionine. In addition, the expression levels or the enzymatic activities of proteins involved indirectly in biodesulfurization might also be affected by alterations in the metabolic profile of recombinants. Examples are the oxidoreductase DszD, relevant cofactors (NADH, FMN), and proteins involved in the import of DBT.

In the presence of cysteine as the sole sulfur source, a functional, forward transsulfuration pathway can produce homocysteine, a precursor for MetE/MetH-mediated methionine biosynthesis. Alternatively, cysteine can be converted to sulfide by a cysteine desulfhydrase. Based on the calculated *C*_max_ values, cysteine supplementation at 0.1 and 1 mM repressed the biodesulfurization activity of the wt and mutants, whereas at the same time it allowed or even promoted growth. In contrast, a high concentration of the amino acid (10 mM) had significant adverse effects on biomass concentration maxima of wt and Δ*cbs* strains but not the Δ*metB* strain. Cysteine-mediated toxicity at high concentrations was reported to inhibit yeast growth (61). The fact that the Δ*metB* strain remains mostly insensitive to cysteine abundance in the culture medium, concomitant with slightly elevated (although nonsignificant) μ_max_ values for lower cysteine content, could indicate a more prominent cysteine deficiency, caused by the absence of MetB. The biodesulfurization activities exhibited by the recombinant strains grown in the presence of repressive sulfur sources are in line with the results reported by Tanaka et al. (39) for R. erythropolis KA2-5-1, as sulfate and methionine did not seem to be directly involved in the repression system, in contrast to cysteine. Therefore, cysteine supplementation, even at 0.1 mM, probably constitutes an impeding factor for DBT biodesulfurization, regardless of the reverse transsulfuration pathway functionality. Notably, cysteine is known to regulate the expression of genes involved in sulfur assimilation by modulating the formation of a complex between CysK and CysE or between CysK and the transcription factor CymR (see below) (75).

**FIG 10:**
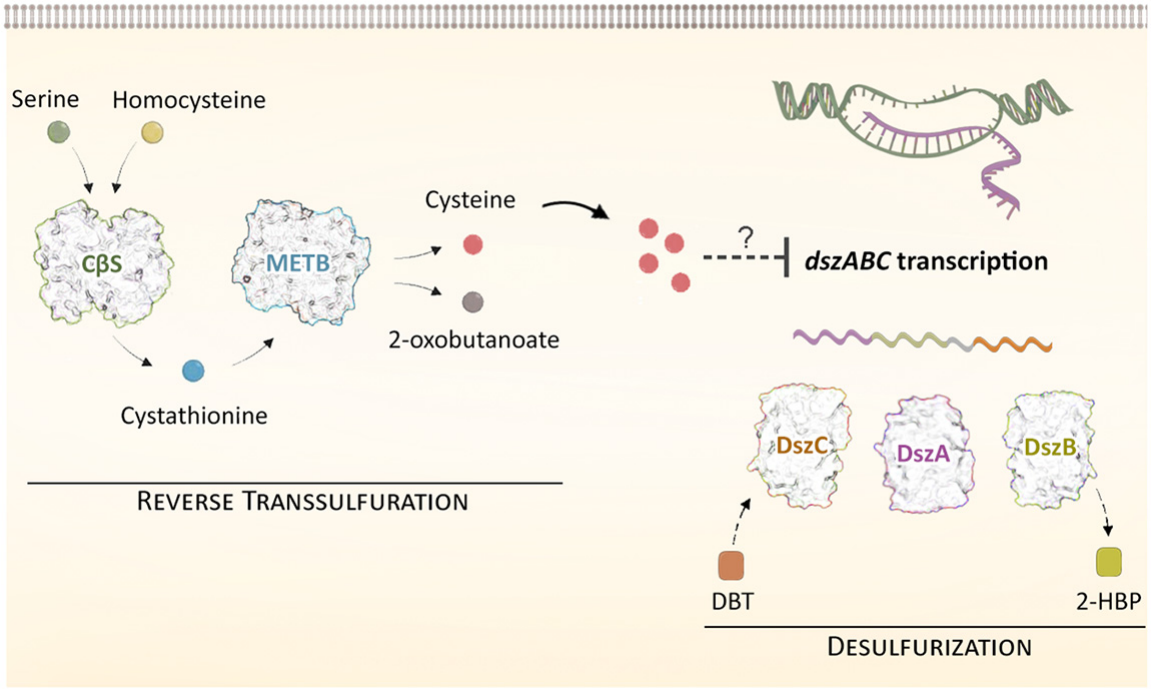
Proposed model illustrating the role of CβS and MetB (acting as CγL) in the regulation of desulfurization activity in Rhodococcus qingshengii IGTS8. Sulfate or methionine addition in the culture medium most likely necessitates reverse transsulfuration metabolic reactions as the primary route for cysteine biosynthesis. Fine-tuning of sulfur assimilation via intracellular cysteine levels is a common theme in bacterial species, where it seems to have evolved as a cellular mechanism to control gene expression appropriately, based on the available sulfur source type and abundancy. Narrow alterations in the free cysteine pool are suspected to exert an effect (directly or indirectly) on *dszABC* gene expression, leading to lack of biodesulfurization activity. Deletion of *cbs* or *metB* abolishes *dsz* repression in the presence of selected sulfur sources, such as sulfate and methionine, thus leading to detectable transcript levels and biodesulfurization activity.

As evidenced by transcript level comparison for wt, Δ*cbs* and Δ*metB* strains, CβS and MetB exert an effect on *dszABC* gene expression, in response to the supplementation of different sulfur sources. Under physiological conditions, the reverse transsulfuration enzymes regulate cysteine biosynthesis and likely promote a slight increase of the free cysteine pool, when either sulfate or methionine is used as the sole sulfur source. Deletions of the two genes might lead to reduction but not depletion of intracellular cysteine levels, promoting a global fine-tuning of sulfur starvation-induced proteins expression. This, in turn, could eventually allow efficient *dszABC* expression in Δ*cbs* and Δ*metB* strains, under sulfate- or methionine-rich conditions, given that expression of sulfur assimilation genes is widely modulated in response to sulfur source availability (Fig. 10) (4, 76). This hypothesis is in line with the complete lack of biodesulfurization activity and the nondetectable *dsz* gene expression observed for the wt and knockout strains in the presence of exogenously provided cysteine as the sole sulfur source.

The mechanistic details of cysteine effects on *dsz* repression have not been elucidated in *R. qingshengii* IGTS8; therefore, direct binding of the metabolite to a homologue of the *dsz* operon repressor WhiB1 cannot be excluded (8). However, it is possible that cysteine promotes CysK-CymR complex formation, via inhibition of the CysE-CysK bienzyme complex (74, 75). Importantly, homologues of CysK, CysE, and CymR are harbored within the IGTS8 genome (BLAST analysis; data not shown). Once activated, CymR (77, 78) or some other global regulator of cysteine metabolism could have a direct repressive effect on the *dsz* operon or modulate its regulators, DszGR and WhiB1 (6–8). In addition to DszGR, the general DNA binding protein IHF was also shown to be necessary for *P_dsz_* promoter activity, but its role has not been validated via knockout of the *mihF* gene in *R. qingshengii* IGTS8 or *Gordonia* sp. (9). Interestingly, an initial study postulated the existence of a ligand binding site in DszGR for sodium sulfate, leading to a change in the structure of the protein *in vitro*. This approach, however, did not conclusively show a direct role for sulfate in *dsz* operon repression for *Rhodococcus* strain IGTS8 (7). Based on our findings, *dszABC* gene expression is no longer repressed in the presence of 1 mM sulfate, once CβS or MetB is depleted. Regulation might differ slightly from one genus (*Gordonia*) to another (*Rhodococcus*), given that regulatory sequences of the *P_dsz_* promoter regions are only partially conserved (10). Moreover, we cannot exclude the possibility that other regulators are present within the IGTS8 genome and thus could have dominant or synergistic effects on *dsz* operon regulation *in vivo*. Some examples are the CymR homologue described in detail above and the global CysB regulator (79). Taken together, these results show that several mechanistic details remain to be elucidated, offering valuable information on biodesulfurization regulation.

In conclusion, our approach provides significant insights into the metabolic engineering of sulfur metabolism in Rhodococcus qingshengii IGTS8 without manipulation of the 4S pathway genes and reveals an important role for CβS and MetB in the global regulation of Dsz-mediated sulfur acquisition from organosulfur compounds, such as DBT. From our own observations and the available data in the literature, we propose the involvement of both enzymes in the reverse transsulfuration pathway of Rhodococcus qingshengii IGTS8, we highlight an important, yet unexplored, role for cysteine in *dsz* gene expression and biodesulfurization activity, and we validate the necessity of intact *cbs* and *metB* loci for the orchestration of *dsz-*mediated sulfur assimilation, in response to sulfur source availability.

## MATERIALS AND METHODS

### Strains, growth conditions, and plasmids.

The bacterial strains and plasmids used in this study are listed in Table 1. Rhodococcus qingshengii IGTS8 was obtained from ATCC (53968; formerly called Rhodococcus rhodochrous and R. erythropolis). Escherichia coli DH5a and S17-1 were used for cloning and conjugation purposes, respectively. Rhodococcus qingshengii strains were routinely grown in Luria-Bertani peptone (LBP) broth (1% [wt/vol] Bacto peptone, 0.5% [wt/vol] yeast extract, and 1% [wt/vol] NaCl) at 30°C with shaking (180 to 200 rpm) or on LBP agar plates at 30°C. E. coli strains were grown in LB medium (1% [wt/vol] Bacto tryptone, 0.5% [wt/vol] yeast extract, and 1% [wt/vol] NaCl) at 37°C with shaking (180 to 200 rpm) or on LB agar plates at 37°C. Kanamycin (50 μg/mL) was used for plasmid selection in E. coli. Kanamycin (200 μg/mL) and nalidixic acid (10 μg/mL) were used to select *R. qingshengii* transconjugants in the culture media. Counterselection was performed on no-salt LBP (NSLBP) plates with 10% (wt/vol) sucrose.

**TABLE 1:** Bacterial strains and plasmids used in this study

Strain or plasmid	Description	Source or reference
Strains		
*R. qingshengii* IGTS8 (wt)	DBT-degrading bacterium, wt strain	ATCC 53968
Δ*cbs* strain	Genetically engineered IGTS8 strain with *cbs* deletion	This study
Δ*metB* strain	Genetically engineered IGTS8 strain with *metB* deletion	This study
E. coli DH5α	F^−^ Δ(*lacZYA*-*argF*)*U169 hsdR17*(r_K_^−^ m_K_^+^) *recA1 endA1 relA1*	Laboratory stock
E. coli S17-1	*recA pro hsdR* RP4-2-Tc::MuKm::Tn*7*	ATCC 47055

Plasmids		
pK18mobsacB	Suicide vector derived from plasmid pK18; RP4 *mob*, *sacB*, Kan^r^	81
pIGTS8cbs	Derived from pK18mobsacB for *cbs* deletion; RP4 *mob*, *sacB*, Kan^r^	This study
pIGTS8metB	Derived from pK18mobsacB for *metB* deletion; RP4 *mob*, *sacB*, Kan^r^	This study

For biodesulfurization studies, *R. qingshengii* wt and recombinant strains were grown on a sulfur-free chemically defined medium (CDM) containing 3.8 g NaH_2_PO_4_·H_2_O, 3.25 g Na_2_HPO_4_·7H_2_O, 0.8 g NH_4_Cl, 0.325 g MgCl_2_·6H_2_O, 0.03 g CaCl_2_·2H_2_O, 8.5 g NaCl, 0.5 g KCl, 1 mL metal solution, and 1 mL of vitamin solution in 1 L of distilled water (pH 7.0). The metal solution contained the following (per liter of distilled water): Na_2_-EDTA, 5.2 g; FeCl_2_·4H_2_O, 3 mg; H_3_BO_3_, 30 mg; MnCl_2_·4H_2_O, 100 mg; CoCl_2_·6H_2_O, 190 mg; NiCl_2_·6H_2_O, 24 mg; CuCl_2_, 0.2 mg; ZnCl_2_, 0.5 mg; Na_2_MoO_4_·2H_2_O, 36 mg; Na_2_WO_4_·2H_2_O, 8 mg; and Na_2_SeO_3_·5H_2_O, 6 mg. The vitamin solution contained the following (per liter of distilled water): calcium pantothenate, 50 mg; nicotinic acid, 100 mg; *p-*aminobenzoic acid, 40 mg; and pyridoxal hydrochloride, 150 mg. CDM was supplemented with dibenzothiophene (DBT) (0.1 mM) or with dimethyl sulfoxide (DMSO), sulfate, l-methionine, l-cysteine as the sole sulfur source (0.1 or 1 mM), and 0.165 M ethanol, 0.055 M glucose, or 0.110 M glycerol as the carbon source (0.33 M carbon), depending on the experiment. pK18mobsacB (Life Science Market, Europe) was used as a cloning and mobilization vector.

### Enzymes and chemicals.

All restriction enzymes were purchased from TaKaRa Bio or Minotech (Lab Supplies Scientific SA, Greece). Chemicals were purchased from Sigma-Aldrich (Kappa Lab SA, Greece) and AppliChem (Bioline Scientific SA, Greece). Conventional and high-fidelity PCR amplifications were performed using KAPA *Taq* DNA and Kapa HiFi polymerases, respectively (Kapa Biosystems, Roche Diagnostics, Greece). All oligonucleotides were purchased from Eurofins Genomics (Vienna, Austria) and are listed in Table S1.

10.1128/mbio.00754-22.5 TABLE S1 Oligonucleotides used in this study. Download Table S1, PDF file, 0.1 MB. Copyright © 2022 Martzoukou et al. 2022 Martzoukou et al. https://creativecommons.org/licenses/by/4.0/ This content is distributed under the terms of the Creative Commons Attribution 4.0 International license.

### Construction of knockout strains.

The genomic DNA of *Rhodococcus* strain IGTS8 was isolated using the NucleoSpin tissue DNA extraction kit (Macherey-Nagel, Lab Supplies Scientific SA, Greece) according to the manufacturer’s instructions. The online software BPROM was used for bacterial promoter prediction (http://www.softberry.com/berry.phtml?topic=bprom&group=programs&subgroup=gfindb) (83). Unmarked, precise gene deletions of cystathionine β-synthase (*cbs*; IGTS8_peg3012) or cystathionine γ-lyase/synthase (*metB*; IGTS8_peg3011) were created using a two-step allelic exchange protocol (80). Upstream and downstream flanking regions of the *cbs* gene of strain IGTS8 were amplified and cloned into the pK18mobsacB vector (81), using the primer pairs cbsUp-F/Up-R and cbsDown-F/Down-R, respectively, yielding plasmid pIGTS8*cbs*. Similarly, for the flanking regions of the *metB* gene, the primer pairs metBUp-F/Up-R and metBDown-F/Down-R were used to construct plasmid pIGTS8*metB*. Plasmid preparation and DNA gel extraction were performed using the NucleoSpin plasmid kit and the NucleoSpin Extract II kit (Macherey-Nagel, Lab Supplies Scientific SA, Greece). E. coli S17-1 competent cells were transformed with each of the modified plasmids. *R. qingshengii* IGTS8 knockouts were created after conjugation (82) with E. coli S17-1 transformants, using a two-step homologous recombination (HR) process (Fig. S1). Following the first crossover event, sucrose-sensitive and kanamycin-resistant IGTS8 transconjugants were grown in LB overnight with shaking (180 rpm) to induce the second HR event. Recombinant strains were grown on selective media containing 10% (wt/vol) sucrose and tested for kanamycin sensitivity, to remove incomplete crossover events. The gene deletions Δ*cbs* and Δ*metB* were identified with PCR and confirmed by DNA sequencing of the PCR products (Eurofins-Genomics, Vienna, Austria), using the external primer pairs cbs-5F-check/cbs-3R-check and metB-5F-check/metB-3R-check for Δ*cbs* and Δ*metB*, respectively.

### Growth and desulfurization assays.

For inoculum preparation, cells were harvested from LBP plates in Ringer’s buffer, pH 7.0, and centrifuged at 3.000 rpm for 10 min, and medium was discarded. The wash process was repeated twice. Pellet was resuspended in 0.5 mL of the same buffer, and necessary dilutions were performed for measurements of optical density at 600 nm (OD_600_) and inoculum standardization. Biomass concentration, expressed as dry weight of cells, was estimated by measurement of optical density at 600 nm with a Multiskan GO microplate spectrophotometer (Thermo Fisher Scientific, Waltham, MA, USA), and calculations were based on an established calibration curve. Initial inoculum concentration was determined, and an initial biomass concentration of 0.045 to 0.055 g/L was applied for each growth condition.

For growth studies and resting cells biodesulfurization assays (Fig. 4 to 8; Fig. S3 and S4), wt and/or recombinant strains were inoculated as described above and grown in CDM with different carbon and sulfur source types and concentrations (described in the respective figure legends). Growth took place in 96-well cell culture plates, with the use of 17 identical well cultures per strain and condition (F-bottom plates; Greiner Bio-One, Fisher Scientific, USA) with 150 μL working volume in thermostated plate shakers at 30°C and 600 rpm (PST-60HL; BioSan, Pegasus Analytical SA, Greece). Biomass concentration at all time points was calculated as described above.

10.1128/mbio.00754-22.4 FIG S4 Linearity of biodesulfurization assay with respect to biomass concentration. Graph showing the linear relationship between the concentration of detected 2-HBP (in micromolar units) and the concentration of biomass (in grams per liter) used in the biodesulfurization assay. The line corresponds to linear regression for the plotted points. Assay time, 1 h. Download FIG S4, TIF file, 0.1 MB. Copyright © 2022 Martzoukou et al. 2022 Martzoukou et al. https://creativecommons.org/licenses/by/4.0/ This content is distributed under the terms of the Creative Commons Attribution 4.0 International license.

For growth and desulfurization studies in the presence of DBT, *R. qingshengii* IGTS8 wt, Δ*cbs*, and Δ*metB* strains were grown in CDM with supplementation with 0.1 mM DBT (from a 100 mM ethanol stock) as the sole sulfur source and ethanol at a final concentration of 0.165 M as the sole carbon source. Three identical 100-mL flasks with a 20-mL working volume were used for each strain.

For modeling microbial growth, a simple unstructured logistic kinetic model was employed:

$$\frac{\text{d}C_{X}}{\text{d}t}={\text{μ}}_{\text{max}}\cdot C_{X}\cdot \left(C_{X}^{\text{max}}\;-\;C_{X}\right)$$

where *C_X_* is the biomass concentration (in grams per liter), $C_{X}^{\text{max}}$ is the final biomass concentration (in grams per liter), and μ_max_ is the apparent maximum specific growth rate (per hour). A nonlinear regression (SigmaPlot, version 12) routine was used to determine the model parameters $C_{X}^{\text{max}}$ and μ_max_ for each experimental data set (*C_X_* versus *t*) using the integrated form of the above equation. The minimization of the sum of squared residuals was used as the convergence criterion.

Resting-cell biodesulfurization assays were performed simultaneously with the growth studies, using the cells of the corresponding 96-well-plate cultures. The contents of 2 to 4 identical well cultures were harvested at early exponential, mid-exponential, and late exponential phases and centrifuged at 3.000 rpm for 10 min, and the medium was discarded. Pellets were washed with an S-free buffer of pH 7.0 (Ringer’s), and cells were resuspended in 0.45 mL of 50 mM HEPES buffer, pH 8.0. Suspensions were separated into three equal-volume aliquots (0.15 mL) in Eppendorf tubes. Next, 0.15 mL of a 2 mM DBT solution prepared in the same buffer was added to each tube, and the desulfurization reaction took place with shaking (1,200 rpm) for 30 min at 30°C in a thermostated Eppendorf shaker (Thermo Shaker TS-100, Boeco, Germany). The reaction was terminated with the addition of an equal volume (0.3 mL) of acetonitrile (Labbox Export, Kappa Lab SA, Greece) and vigorous vortexing. Suspensions were centrifuged (14.000 × *g*; 10 min), and 2-HBP produced was determined in the collected supernatant by high-performance liquid chromatography (HPLC). One of the tubes to which the 0.3 mL acetonitrile was added immediately after DBT addition (*t* = 0) was used as a blank. Desulfurization capability was expressed as units per milligram of cells (dry weight), where 1 unit corresponds to the release of 1 nmol of 2-HBP per hour. The linearity of the above-described assay with respect to biomass concentration has been verified for up to a 2-h reaction time and up to 100 μΜ 2-HBP produced in the sample (Fig. S4). According to the procedure described above, every technical replicate (*n* = 2) corresponds to the biological mean of 2 to 5 replicate wells.

### HPLC analysis.

HPLC was used to quantify 2-HBP and DBT. The analysis was performed on an Agilent HPLC 1220 Infinity LC System, equipped with a fluorescence detector (FLD). A C_18_ reversed-phase column (Poroshell 120 EC-C18; 4 μm, 4.6 by 150 mm; Agilent) was used for the separation. The elution profile (at 1.2 mL/min) consisted of 4 min isocratic elution with 60/40 (vol/vol) acetonitrile-H_2_O, followed by a 15-min linear gradient to 100% acetonitrile. Fluorescence detection was performed with excitation and emission wavelengths of 245 nm and 345 nm, respectively. Quantification was performed using appropriate calibration curves with the corresponding standards (linear range, 10 to 1,000 ng/mL).

### Extraction of total RNA.

*R. qingshengii* IGTS8 wt, Δ*cbs*, and Δ*metB* strains were grown in CDM containing DMSO, MgSO_4_, methionine, or cysteine as the sole sulfur source (1 mM), as described above in “Growth and desulfurization assays.” Ethanol was used as a carbon source to a final concentration of 0.165 Μ (0.33 M carbon). Cells were harvested in mid-exponential phase and incubated with lysozyme (20 mg/mL) for 2 h at 25°C. Total RNA isolation was performed using a NucleoSpin RNA kit (Macherey-Nagel, Lab Supplies Scientific SA, Greece) according to manufacturer guidelines. RNA samples were treated with DNase I as part of the kit procedure to eliminate any genomic DNA contamination. RNA concentration and purity were determined at 260 and 280 nm using a μDrop plate with a Multiskan GO microplate spectrophotometer (Thermo Fisher Scientific, Waltham, MA, USA), while RNA integrity was evaluated by agarose gel electrophoresis.

### First-strand cDNA synthesis.

Reverse transcription took place in a 20-μL reaction mixture containing 500 ng total RNA template, a mixture of deoxynucleoside triphosphates (dNTPs) at 0.5 mM, 200 U SuperScript II reverse transcriptase (Invitrogen, Antisel SA, Greece), 40 U RNaseOUT recombinant RNase inhibitor (Invitrogen, Antisel SA, Greece), and a 4 μM concentration of random hexamer primers (TaKaRa Bio, Lab Supplies Scientific SA, Greece). Reverse transcription was performed at 42°C for 50 min, followed by enzyme inactivation at 70°C for 15 min. The concentration of cDNA was determined using a μDrop plate with a Multiskan GO microplate spectrophotometer (Thermo Fisher Scientific, Waltham, MA, USA).

### qPCR.

Quantitative real-time PCR (qPCR) assays were performed on the 7500 real-time PCR system (Applied Biosystems, Carlsbad, CA) using SYBR green I dye for the quantification of *dszA*, *dszB*, *dszC*, *cbs*, and *metB* transcript levels. Specific primers were designed based on the published sequences of the IGTS8 desulfurization operon (GenBank accession no. U08850.1 for *dszABC*) and IGTS8 chromosome (GenBank accession no. CP029297.1 for *cbs*, *metB*, and *gyrB*) and are listed in Table S1. The gene-specific amplicons generated were 143 bp for *dszA*, 129 bp for *dszB*, 152 bp for *dszC*, 226 bp for *cbs*, 129 bp for *metB*, and 158 bp for *gyrB*. The 10-μL reaction mixture included 5 μL Kapa SYBR Fast universal 2× qPCR master mix (Kapa Biosystems, Lab Supplies Scientific SA, Greece), 5 ng of cDNA template, and a 200 nM concentration of each specific primer. The thermal protocol was initiated at 95°C for 3 min for polymerase activation, followed by 40 cycles of denaturation at 95°C for 15 s, and primer annealing and extension at 60°C for 1 min. Following amplification, melt curve analyses were carried out to distinguish specific amplicons from nonspecific products and/or primer dimers. All qPCRs were performed using two technical replications for each tested sample and target, and the average CT of each duplicate was used in quantification analyses, according to the 2^−ΔΔ^*^CT^* relative quantification (RQ) method. The DNA gyrase subunit B (*gyrB*) gene from strain IGTS8 was used as an internal reference control for normalization purposes. A cDNA sample derived from *R. qingshengii* IGTS8 grown on 1 mM DMSO for 66 h was used as our assay calibrator. All qPCR procedures and results are reported according to the MIQE (minimum information for publication of quantitative real-time PCR experiments) guidelines. See also Table S3.

10.1128/mbio.00754-22.7 TABLE S3 Data sheet for MIQE guidelines. Download Table S3, PDF file, 0.3 MB. Copyright © 2022 Martzoukou et al. 2022 Martzoukou et al. https://creativecommons.org/licenses/by/4.0/ This content is distributed under the terms of the Creative Commons Attribution 4.0 International license.

### Statistical analysis.

Specific growth rates (μ_max_) and maximum biomass concentrations (*C*_max_) were calculated as described above in “Growth and desulfurization assays.” The results of growth studies (μ_max_ and *C*_max_ values) were statistically analyzed by performing one-sample *t* tests for each strain and each medium composition (confidence interval was set to 95%; *n* = 12 to 17). For comparison of growth kinetic parameters between different strains or medium compositions, an unpaired *t* test or one-way analysis of variance (ANOVA) with Tukey’s multiple-comparison test was performed depending on the number of compared columns (confidence interval was set to 95%; *n* = 12 to 17). Statistical significance is described in the corresponding sections in Results.

To compare biodesulfurization activities (units per milligram of cells [dry weight]) for results shown in Fig. 4, one-way ANOVA with Tukey’s multiple-comparison test was performed between different growth conditions (glucose versus glycerol versus ethanol as the sole carbon source). For the statistical analyses of biodesulfurization activities in Fig. 5 to 8, one-way ANOVA with Tukey’s multiple-comparison test was performed comparing results for the wt and each of the recombinant strains for the same growth conditions (i.e., same carbon and sulfur sources) at the same time point (20, 45, and 65 h). *P* values are indicated in the corresponding figures. Our analysis also included comparison of different concentrations of the same sulfur source used to supplement the same strain. *P* values are mentioned in Results. The confidence interval was set to 95% in all cases.

For results shown in Fig. 9, one-way ANOVA with Tukey’s multiple-comparison test was performed, to compare transcription levels of wt and each of the recombinant strains. Two biological and two technical replicates were used (see also Table S3). The software used for statistical analyses was GraphPad Prism 6.01.
