# Supplementary figures and images for: Interplay between Sulfur Assimilation and Biodesulfurization Activity in Rhodococcus qingshengii IGTS8: Insights into a Regulatory Role of the Reverse Transsulfuration Pathway
Source: mBio. 2022 Jul 20;13(4):e00754-22. doi: 10.1128/mbio.00754-22 (PMC9426449; doi:10.1128/mbio.00754-22)

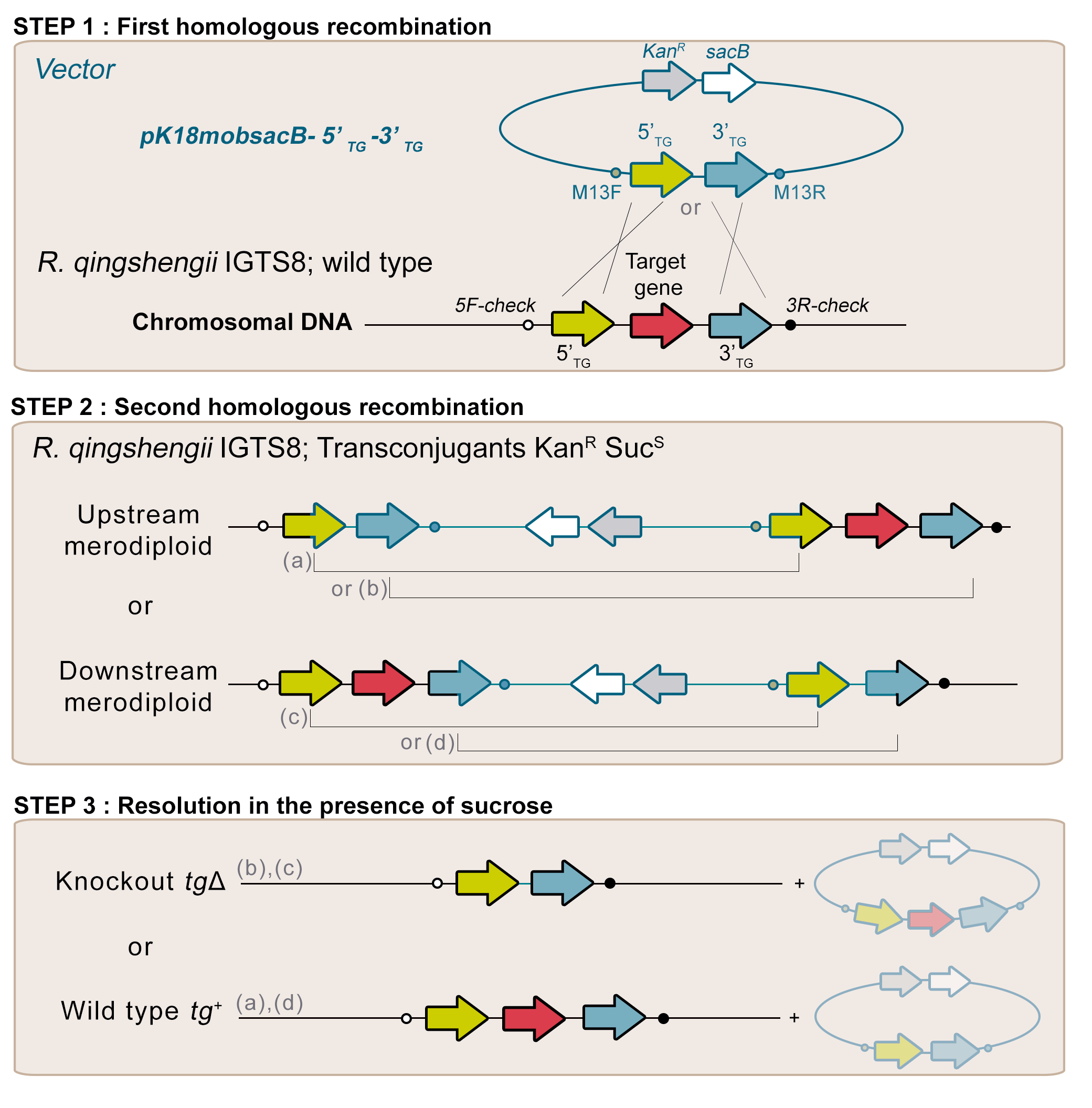

Supplement: FIG S1 [file mbio.00754-22-s0001.tif]

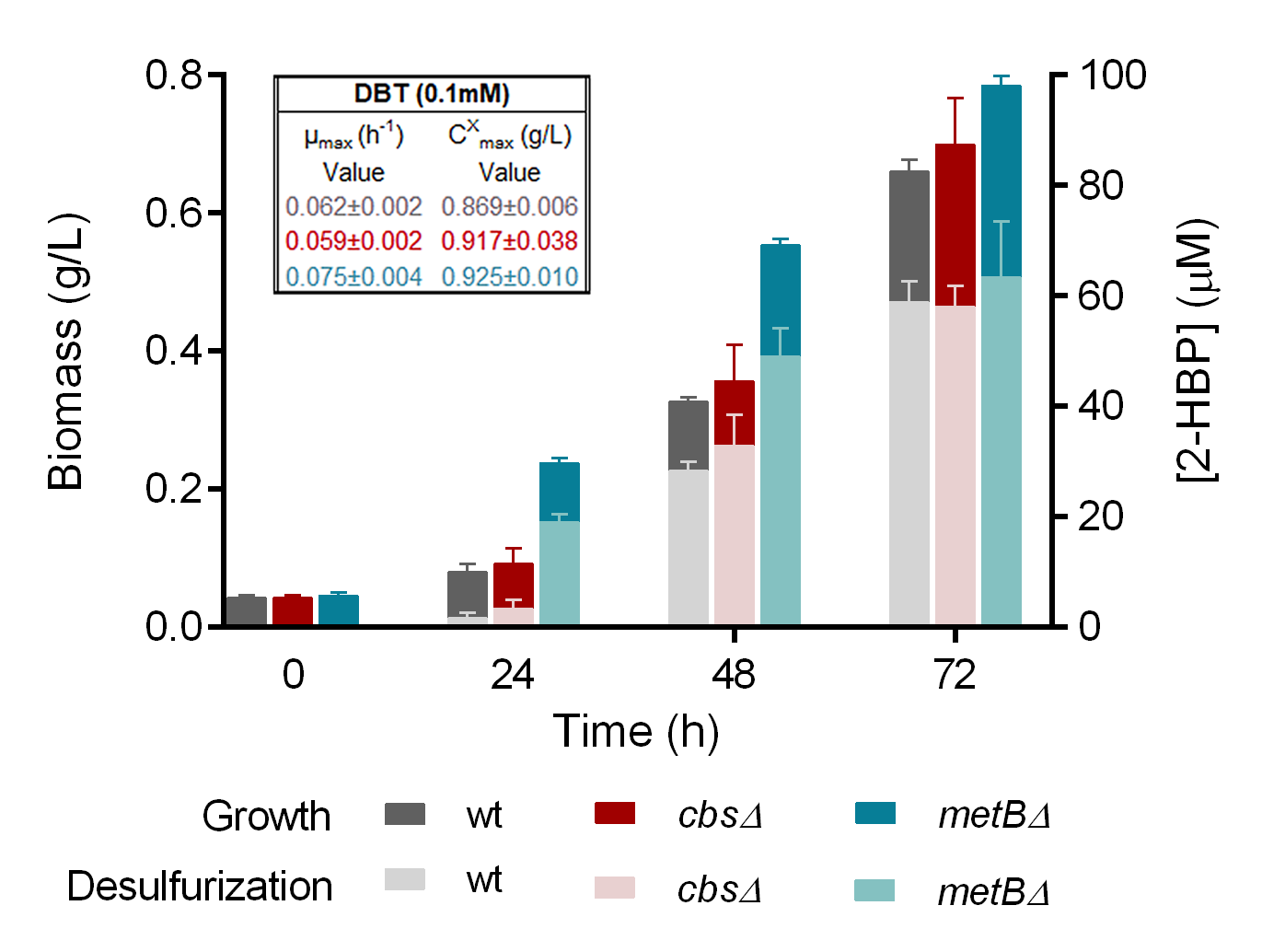

Supplement: FIG S2 [file mbio.00754-22-s0002.tif]

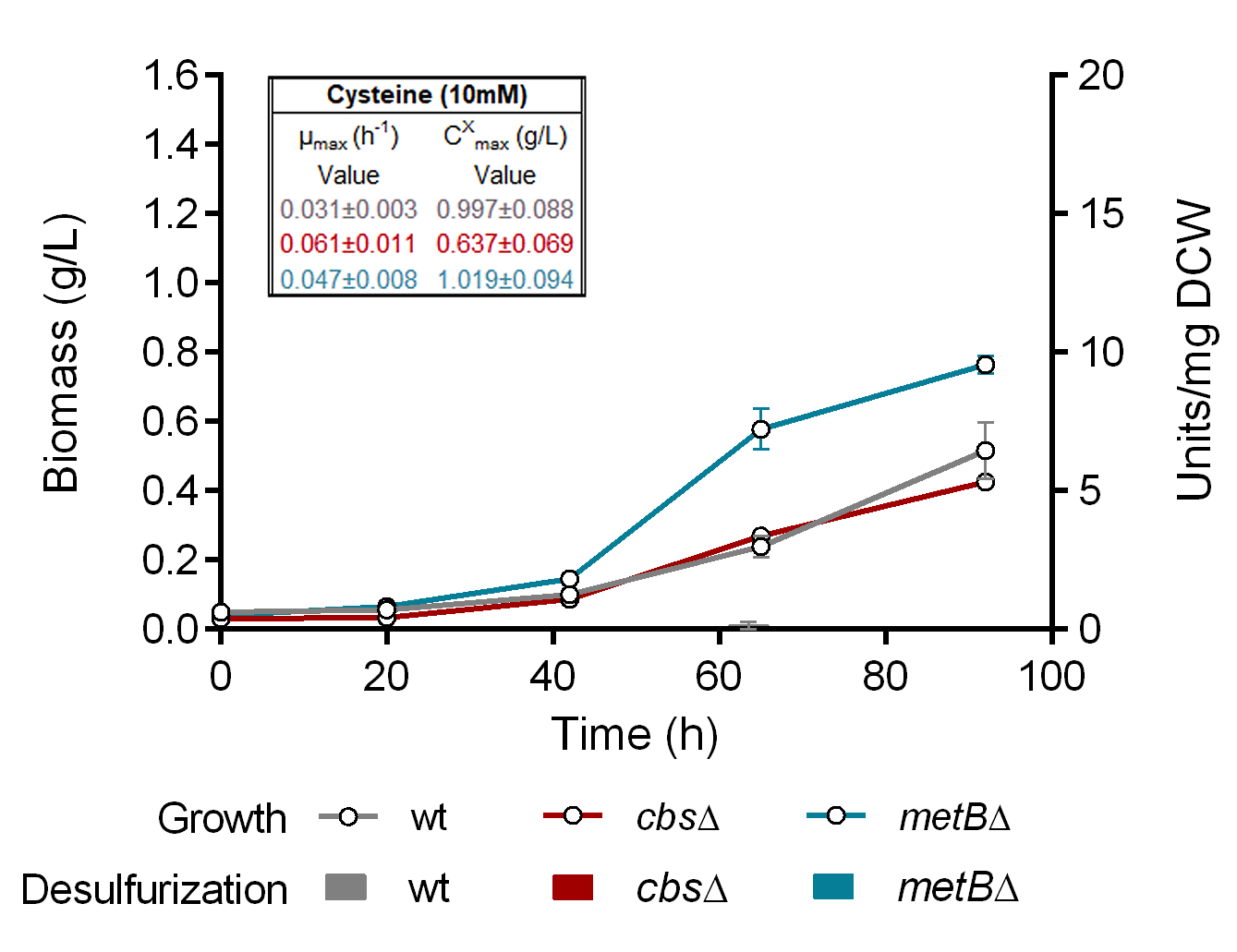

Supplement: FIG S3 [file mbio.00754-22-s0003.tif]

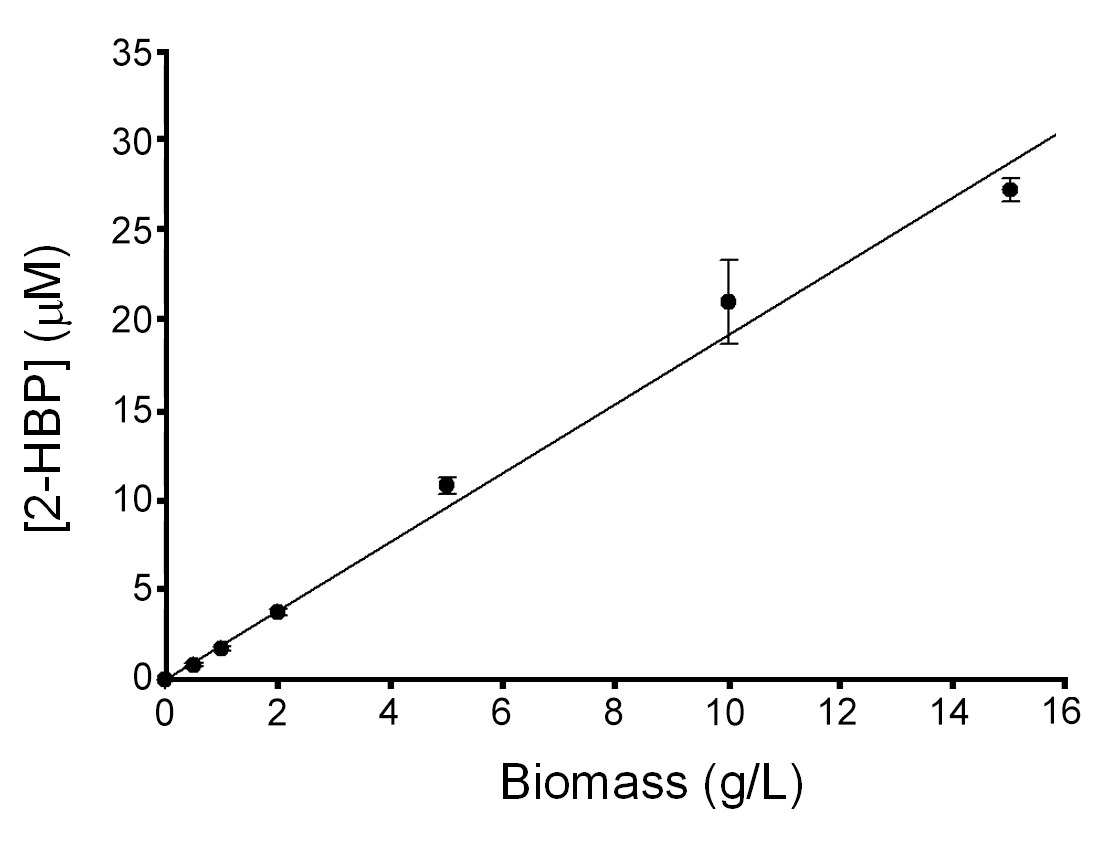

Supplement: FIG S4 [file mbio.00754-22-s0004.tif]
