# Supplementary material for: Interplay between Sulfur Assimilation and Biodesulfurization Activity in Rhodococcus qingshengii IGTS8: Insights into a Regulatory Role of the Reverse Transsulfuration Pathway
Source: mBio. 2022 Jul 20;13(4):e00754-22. doi: 10.1128/mbio.00754-22 (PMC9426449; doi:10.1128/mbio.00754-22)
Supplement: TEXT S1 [file mbio.00754-22-s0008.pdf]

Supplementary Information

DNA Sequences of cbs and metB genes

Color code: Predicted promoter RBS Start codon End codon

>*R. qingshengii* IGTS8 wild-type

CGTGGCCACCGACGCTTTCAGTCCGGTTCGACTCACACTTGCCAATCTAGCCGGATTGCACCACCTTCGCCCGACGTCCGCGCG
CACCGTTTTCTTTCATCTGGGAACATGGACGAATGCGCATCGCGGAACACGTCGTCGACCTCATCGGCAACACCCCACTCGTCA
AGCTCACGTCCGTCACGGGCGAAAACTTCGGCACGGTGGCAGCCAAGGTCGAATACCTCAACCCTGGCGGTAGCTCCAAGGA
CCGCATCGCCGTCAAGATGATCGACGCAGCCGAGGCTTCGGGTGAACTGAAGCCGGGCGGCACCATCGTCGAGCCGACCTCG
GGCAACACCGGAATCGGCTTGGCGCTCGTGGCGCAAAAGCGTGGATACAAGTGCGTTTTCGTCTGCCCAGACAAGGTCAGCG
AAGACAAGCGCAACGTCCTGCGCGCCTACGGCGCCGAGGTGGTCGTGTGCCCCACGGCCGTCGCGCCGGACAACCCGAACAG
CTACTACAGCGTCTCCGACCGCCTCACCCGTGAGATCCCCGGCGCCTGGAAGCCCAACCAGTACTCCAACCCGGGCGGACCGG
AGAGCCACTACGAGACCACCGGCCCCGAGATCTGGGCCGACACCGACGGCAAGATCACGCACTTCGTCGCGGGCGTCGGCAC
CGGCGGCACCATCACCGGCACCGGCCGCTACCTCAAGGAAGTTTCGGGCGGCAAGGTCAAGGTCATCGGCGCAGACCCCGAG
GGCTCCGTCTACTCCGGCGGCACCGGTCGTCCGTATCTGGTCGAAGGCGTCGGCGAAGACTTCTGGCCGTCGGCATACGACCC
GTCCATCCCGGACGAGATCATCGCCGTCTCGGATGCCGATTCCTTCGAGATGACGCGTCGCCTCGCCCGCGAGGAGGGACTGC
TGGTCGGCGGTTCCTGCGGTATGGCCGTCGTCGCTGCCCTCGAAGTAGCCAAGCGTGAGGGCCCCGACGCCCTGATCGTCGTC
CTCCTCCCGGACGGTGGCCGCGGCTACCTGTCCAAGATCTTCAACGATCAGTGGATGGCGTCGTACGGATTCCTGCGCACCCC
ACTCGACGGCAAGACCAACGTCCCCACCGTCGGCGACGTTCTGCGTGGCAAGTCGGGCGAGCTGCCGGACCTCGTTCACACCC
ACCCGTCGGAGACCCTGCGCGACGCGATCGAGATCCTGCGCGAGTACGGCGTGTCCCAGATGCCCGTCGTGGGTGCGGAACC
GCCGGTCATGGCAGGCGAGGTCGCCGGAAGTGTGTCCGAGCGGGATCTGCTGAGCGCGGTCTTCGAAGGCCGTGCGCACCTT
GCGGATTCCGTCGAGAAGCACATGAGCAAGCCGTTCCCGCTCATCGGTTCCGGCGAGCCGGTCTCGGCTGCCACGAAGGCGC
TCGGGGACACCGACGCACTCATGGTCGTCGACGACGGCAAGCCGGTCGGTGTCATCACGCGCCACGATCTCCTGGGTTTCCTG
AGTTCGGATTCCTGAGTTCGGATTCCTGCGCCGGCACCGGCGCCATTAGGCTGGGGACCATGAGTGAGCAACGCAGCAAGGC
CGACAACATCAGCTGGCAGGGTTTCTCCACGAAAGCGGTGCATGCGGGTTACGAACCCGATCCGCTGACCGGTGCGGTCAAC
GTGCCGATCTACGCGAGTTCCACCTTCGCCCAGGACGGCGTCGGTGGAATGCGCAGTGGCTTCGAGTACGCGCGAACGGGTA
ACCCCACCCGTCGCCCACTCGAGGCGAACCTCGCAGCGCTCGAATCCGGAACCTACGGCCGAGCGTTCAGTTCGGGAATGGC
GGCCACCGACTGCCTGCTCCGTTCGGTACTTCGACCGGGCGATCACCTGGTCATCCCCGACGACGCGTACGGCGGAACCTTCC
GCCTCATCGACAAGGTCTTCACGCAGTGGGGGATCGAGTACACCCCGGCTCCGGTGTCCGACGTCGATGCGGTGCGCGCGGC
GATGAAGTCCAACACCAAGCTGGTCTGGGTCGAGACGCCCACGAACCCGCTGCTCAATATCGGTGACATCGAGCTCCTTGCCG
AGGTCGCCCACGAGGGCAACGCAAAGATCGTGGTGGACAACACCTTCGCCTCGCCGTACCTGCAGCAGCCGCTGCAGCTCGG
TGCCGACATCGCGCTGCACTCCACGACCAAGTACATCGGCGGACACTCCGACGTGGTCGGTGGCGCTCTCGTCTGCAACGACG
AAGAACTGGACACCGCGTTTGCCTTCCTGCAGAACGGATCCGGCGGCGTTCCCGGCCCGTTCGACGCGTTCCTGACGTTGCGC
GGAATCAAGACACTGGCGCTGCGCATGGAGCGTCACAGTGACAACGCGGAGAAGGTCGTCGAATTGCTCGACGGCCACCCCG
CGGTTGCCGGCGTCATCTACCCCGGACTGGCTTCGCATCCGTCGCACAAGGTCGCGGCCAAGCAGATGCGACGCTTCGGCGGC
ATGATCTCCGTCCGCCTCAAGGGCGGCAAGGCGGCAGCTCTCGACCTGTGCTCGCGCACCGAGATCTTCACGCTGGCAGAATC
TCTCGGCGGAGTCGAATCCCTCATCGAATTCCCCGGTGCGATGACCCACGCCTCGACGGCCGGTTCCGCTCTCGAGGTTCCCGA
CGATCTGGTTCGCCTGTCGGTCGGCATCGAAGACGCCTCCGATCTGGTCGGCGACATCGAGCAAGCGCTGTCCTGATCTTCGC
TCTACAGTAATTCGACTTCGAATCTGCAGTGCGCCTTCACACCTCACGAGGGTGTGGAGGCGCACTTTCTGGAGGCATGC

>*R. qingshengii* IGTS8 *cbsΔ*

CGTGGCCACCGACGCTTTCAGTCCGGTTCGACTCACACTTGCCAATCTAGCCGGATTGCACCACCTTCGCCCGACGTCCGCGCG
CACCGTTTTCTTTCATCTGGGAACATGGACGACTGCAGGTCGACTCTAGAGGATCCCCGGGGTTCGGATTCCTGCGCCGGCAC
CGGCGCCATTAGGCTGGGGACCATGAGTGAGCAACGCAGCAAGGCCGACAACATCAGCTGGCAGGGTTTCTCCACGAAAGC
GGTGCATGCGGGTTACGAACCCGATCCGCTGACCGGTGCGGTCAACGTGCCGATCTACGCGAGTTCCACCTTCGCCCAGGACG
GCGTCGGTGGAATGCGCAGTGGCTTCGAGTACGCGCGAACGGGTAACCCCACCCGTCGCCCACTCGAGGCGAACCTCGCAGC
GCTCGAATCCGGAACCTACGGCCGAGCGTTCAGTTCGGGAATGGCGGCCACCGACTGCCTGCTCCGTTCGGTACTTCGACCGG
GCGATCACCTGGTCATCCCCGACGACGCGTACGGCGGAACCTTCCGCCTCATCGACAAGGTCTTCACGCAGTGGGGGATCGAG
TACACCCCGGCTCCGGTGTCCGACGTCGATGCGGTGCGCGCGGCGATGAAGTCCAACACCAAGCTGGTCTGGGTCGAGACGC
CCACGAACCCGCTGCTCAATATCGGTGACATCGAGCTCCTTGCCGAGGTCGCCCACGAGGGCAACGCAAAGATCGTGGTGGA
CAACACCTTCGCCTCGCCGTACCTGCAGCAGCCGCTGCAGCTCGGTGCCGACATCGCGCTGCACTCCACGACCAAGTACATCG
GCGGACACTCCGACGTGGTCGGTGGCGCTCTCGTCTGCAACGACGAAGAACTGGACACCGCGTTTGCCTTCCTGCAGAACGG
ATCCGGCGGCGTTCCCGGCCCGTTCGACGCGTTCCTGACGTTGCGCGGAATCAAGACACTGGCGCTGCGCATGGAGCGTCACA
GTGACAACGCGGAGAAGGTCGTCGAATTGCTCGACGGCCACCCCGCGGTTGCCGGCGTCATCTACCCCGGACTGGCTTCGCAT
CCGTCGCACAAGGTCGCGGCCAAGCAGATGCGACGCTTCGGCGGCATGATCTCCGTCCGCCTCAAGGGCGGCAAGGCGGCAG
CTCTCGACCTGTGCTCGCGCACCGAGATCTTCACGCTGGCAGAATCTCTCGGCGGAGTCGAATCCCTCATCGAATTCCCCGGTG
CGATGACCCACGCCTCGACGGCCGGTTCCGCTCTCGAGGTTCCCGACGATCTGGTTCGCCTGTCGGTCGGCATCGAAGACGCC
TCCGATCTGGTCGGCGACATCGAGCAAGCGCTGTCCTGATCTTCGCTCTACAGTAATTCGACTTCGAATCTGCAGTGCGCCTTC
ACACCTCACGAGGGTGTGGAGGCGCACTTTCTGGAGGCATGC

>*R. qingshengii* IGTS8 *metBΔ*

CGTGGCCACCGACGCTTTCAGTCCGGTTCGACTCACACTTGCCAATCTAGCCGGATTGCACCACCTTCGCCCGACGTCCGCGCG
CACCGTTTTCTTTCATCTGGGAACATGGACGAATGCGCATCGCGGAACACGTCGTCGACCTCATCGGCAACACCCCACTCGTCA
AGCTCACGTCCGTCACGGGCGAAAACTTCGGCACGGTGGCAGCCAAGGTCGAATACCTCAACCCTGGCGGTAGCTCCAAGGA
CCGCATCGCCGTCAAGATGATCGACGCAGCCGAGGCTTCGGGTGAACTGAAGCCGGGCGGCACCATCGTCGAGCCGACCTCG
GGCAACACCGGAATCGGCTTGGCGCTCGTGGCGCAAAAGCGTGGATACAAGTGCGTTTTCGTCTGCCCAGACAAGGTCAGCG
AAGACAAGCGCAACGTCCTGCGCGCCTACGGCGCCGAGGTGGTCGTGTGCCCCACGGCCGTCGCGCCGGACAACCCGAACAG
CTACTACAGCGTCTCCGACCGCCTCACCCGTGAGATCCCCGGCGCCTGGAAGCCCAACCAGTACTCCAACCCGGGCGGACCGG
AGAGCCACTACGAGACCACCGGCCCCGAGATCTGGGCCGACACCGACGGCAAGATCACGCACTTCGTCGCGGGCGTCGGCAC
CGGCGGCACCATCACCGGCACCGGCCGCTACCTCAAGGAAGTTTCGGGCGGCAAGGTCAAGGTCATCGGCGCAGACCCCGAG
GGCTCCGTCTACTCCGGCGGCACCGGTCGTCCGTATCTGGTCGAAGGCGTCGGCGAAGACTTCTGGCCGTCGGCATACGACCC
GTCCATCCCGGACGAGATCATCGCCGTCTCGGATGCCGATTCCTTCGAGATGACGCGTCGCCTCGCCCGCGAGGAGGGACTGC
TGGTCGGCGGTTCCTGCGGTATGGCCGTCGTCGCTGCCCTCGAAGTAGCCAAGCGTGAGGGCCCCGACGCCCTGATCGTCGTC
CTCCTCCCGGACGGTGGCCGCGGCTACCTGTCCAAGATCTTCAACGATCAGTGGATGGCGTCGTACGGATTCCTGCGCACCCC
ACTCGACGGCAAGACCAACGTCCCCACCGTCGGCGACGTTCTGCGTGGCAAGTCGGGCGAGCTGCCGGACCTCGTTCACACCC
ACCCGTCGGAGACCCTGCGCGACGCGATCGAGATCCTGCGCGAGTACGGCGTGTCCCAGATGCCCGTCGTGGGTGCGGAACC
GCCGGTCATGGCAGGCGAGGTCGCCGGAAGTGTGTCCGAGCGGGATCTGCTGAGCGCGGTCTTCGAAGGCCGTGCGCACCTT
GCGGATTCCGTCGAGAAGCACATGAGCAAGCCGTTCCCGCTCATCGGTTCCGGCGAGCCGGTCTCGGCTGCCACGAAGGCGC
TCGGGGACACCGACGCACTCATGGTCGTCGACGACGGCAAGCCGGTCGGTGTCATCACGCGCCACGATCTCCTGGGTTTCCTG
AGTTCGGATTCCTGAGTTCGGATTCCTGTCTAGAGATCTGGTCGGCGACATCGAGCAAGCGCTGTCCTGATCTTCGCTCTACAG
TAATTCGACTTCGAATCTGCAGTGCGCCTTCACACCTCACGAGGGTGTGGAGGCGCACTTTCTGGAGGCATGC
