# Supplementary material for: Interplay between Sulfur Assimilation and Biodesulfurization Activity in Rhodococcus qingshengii IGTS8: Insights into a Regulatory Role of the Reverse Transsulfuration Pathway
Source: mBio. 2022 Jul 20;13(4):e00754-22. doi: 10.1128/mbio.00754-22 (PMC9426449; doi:10.1128/mbio.00754-22)
Supplement: TABLE S2 [file mbio.00754-22-s0006.pdf]

Table S2. Growth kinetic parameters (μ_{max} and C^{x}_{max}) obtained by fitting of biomass concentration vs. time experimental values to the logistic equation

Strain	Sulfur Source							
	DMSO (0.1mM)		Sulfate (0.1mM)		Methionine (0.1mM)		Cysteine (0.1mM)	
	μ_{max} (h^{-1})	C^{X}_{max} (g/L)	μ_{max} (h^{-1})	C^{X}_{max} (g/L)	μ_{max} (h^{-1})	C^{X}_{max} (g/L)	μ_{max} (h^{-1})	C^{X}_{max} (g/L)
	Value	Value	Value	Value	Value	Value	Value	Value
R.qingshengii IGTS8	0.091±0.002	0.915±0.008	0.089±0.006	1.073±0.032	0.072±0.006	1.080±0.043	0.058±0.006	1.371±0.065
cbsΔ	0.104±0.006	0.684±0.016	0.089±0.007	0.906±0.032	ND	ND	0.054±0.007	1.354±0.088
metBΔ	0.052±0.003	1.286±0.041	0.056±0.007	1.110±0.076	0.030±0.009	1.464±0.345	0.076±0.006	1.234±0.044
	DMSO (1mM)		Sulfate (1mM)		Methionine (1mM)		Cysteine (1mM)	
	μ_{max} (h^{-1})	C^{X}_{max} (g/L)	μ_{max} (h^{-1})	C^{X}_{max} (g/L)	μ_{max} (h^{-1})	C^{X}_{max} (g/L)	μ_{max} (h^{-1})	C^{X}_{max} (g/L)
	Value	Value	Value	Value	Value	Value	Value	Value
R.qingshengii IGTS8	0.093±0.002	0.887±0.099	0.074±0.008	1.226±0.062	0.085±0.006	0.888±0.027	0.060±0.008	1.211±0.083
cbsΔ	0.105±0.006	0.675±0.016	0.086±0.005	1.061±0.031	ND	ND	0.052±0.006	1.315±0.089
metBΔ	0.055±0.003	1.227±0.040	0.058±0.004	1.193±0.046	0.011±0.018	3.135±0.664	0.068±0.005	1.071±0.042
