# Supplementary material for: Interplay between Sulfur Assimilation and Biodesulfurization Activity in Rhodococcus qingshengii IGTS8: Insights into a Regulatory Role of the Reverse Transsulfuration Pathway
Source: mBio. 2022 Jul 20;13(4):e00754-22. doi: 10.1128/mbio.00754-22 (PMC9426449; doi:10.1128/mbio.00754-22)
Supplement: TABLE S1 [file mbio.00754-22-s0005.pdf]

Table S1. Oligonucleotides used in this study

Name	Sequence (5' - 3')
Gene deletions	
cbsUp-F	CGCGAAGCTTGCGAAGGCTTTCCACTGGGTTGCC
cbsUp-R	GCGCCTGCAGTCGTCCATGTTCCCAGATGAAAGA
cbsDown-F	GCGCCCCGGGGTTCGGATTCCTGCGCCGGCACCG
cbsDown-R	CGCGCCCGGGCCGCCCTTGAGGCGGACGGAG
metBUp-F	GCGCAAGCTTGACCGCATCGCCGTCAAGATG
metBUp-R	GCGCTCTAGACAGGAATCCGAACTCAGGAATCC
metBDown-F	GCGCTCTAGAGATCTGGTCGGCGACATCGAG
metBDown-R	GCGCGGATCCCACTTCGTCGAGTGCAAGTTCG
Sequencing	
M13F	AGGGTTTTCCCAGTCACGACGTT
M13R	GAGCGGATAACAATTTCACACAGG
cbs-5F-check	CAGTAACGGTTGACCGTGACACC
cbs-3F-check	CATCGACAAGGTCTTCACGCAGTG
cbs-3R-check	GTTTTCACATTTCAAGCTCACGGCG
metB-5F-check	CGGGGGAGGACCGGCGACGAAC
metB-3R-check	GAAGACGGCTGGCAGATTCAGGTG
qPCR	
QdszAF	CTACTATCCCCCGTATCACGTTG
QdszAR	CGTCGTGTTCCAGATGCTGAT
QdszBF	GCGTATCGACCGGAGCAGT
QdszBR	GCAAGTTGTTGGTGAGCAGGA
QdszCF	GGTTCCACGGACTTCCACAA
QdszCR	GCGATCCCCAGATAGACGTTG
QcbsF	TGGATACAAGTGCGTTTTCGTC
QcbsR	GGTGGTCTCGTAGTGGCTCT
QmetBF	GAGCGTTCAGTTCGGGAATG
QmetBR	GCGTGAAGACCTTGTCGATGA
QgyrBF	GCTGCCCAGAAGTCAGATACA
QgyrBR	TCGACGACCTCCCAAATGAG
