# Supplementary material for: Interplay between Sulfur Assimilation and Biodesulfurization Activity in Rhodococcus qingshengii IGTS8: Insights into a Regulatory Role of the Reverse Transsulfuration Pathway
Source: mBio. 2022 Jul 20;13(4):e00754-22. doi: 10.1128/mbio.00754-22 (PMC9426449; doi:10.1128/mbio.00754-22)
Supplement: TABLE S3 [file mbio.00754-22-s0007.pdf]

Item to check	Importance	Checklist
**EXPERIMENTAL DESIGN**		
Definition of experimental and control groups	E	NA
Number within each group	E	NA
Assay carried out by core lab or investigator's lab?	D	Investigator's lab
Acknowledgement of authors' contributions	D	See author contributions section
**SAMPLE**		
Description	E	Bacterial cultures of wild-type and recombinant *R. qingshengii* IGTS8 strains
Volume/mass of sample processed	D	0.5 - 0.7 mg DCW
Microdissection or macrodissection	E	NA
Processing procedure	E	Materials & Methods section, "Extraction of total RNA"
If frozen - how and how quickly?	E	4 °C, instantly
If fixed - with what, how quickly?	E	NA
Sample storage conditions and duration (especially for FFPE samples)	E	Samples were processed immediately after harvesting
**NUCLEIC ACID EXTRACTION**		
Procedure and/or instrumentation	E	Materials & Methods section, "Extraction of total RNA"
Name of kit and details of any modifications	E	NucleoSpin RNA kit MACHEREY-NAGEL
Source of additional reagents used	D	NA
Details of DNase or RNAse treatment	E	NucleoSpin RNA kit MACHEREY-NAGEL, according to manufacturer's instructions
Contamination assessment (DNA or RNA)	E	Spectrophotometry
Nucleic acid quantification	E	Spectrophotometry
Instrument and method	E	Materials & Methods section, "Extraction of total RNA"
Purity (A260/A280)	D	1.9 - 2.1
Yield	D	50 - 100 ng/µl
RNA integrity method/instrument	E	Materials & Methods section, "Extraction of total RNA"
RIN/RQI or Cq of 3' and 5' transcripts	E	ND
Inhibition testing (Cq dilutions, spike or other)	E	No inhibition detected: calibration curves of the validation qPCR using serial dilutions of assay calibrator
**REVERSE TRANSCRIPTION**		
Complete reaction conditions	E	Materials & Methods section, "First-strand cDNA synthesis"
Amount of RNA and reaction volume	E	500 ng; 20 µL
Priming oligonucleotide (if using GSP) and concentration	E	Random hexamer primers 4 µm
Reverse transcriptase and concentration	E	SuperScript II Reverse Transcriptase 200U
Temperature and time	E	42 °C 50 min
Manufacturer of reagents and catalogue numbers	D	Invitrogen 18064-014
Cqs with and without RT	D	ND
Storage conditions of cDNA	D	-20°C
**qPCR TARGET INFORMATION**		
If multiplex, efficiency and LOD of each assay.	E	No multiplex
Sequence accession number	E	GenBank: U08850.1 for dszABC; CP029297.1 for cbs, metB, gyrB
Amplicon length	E	Materials & Methods section, "Quantitative Real-Time PCR (qPCR)"
In silico specificity screen (BLAST, etc)	E	BLAST
Location of each primer by exon or intron (if applicable)	E	NA
What splice variants are targeted?	E	NA
**qPCR OLIGONUCLEOTIDES**		
Primer sequences	E	Supplementary Information, Table S1, "qPCR" section
RTPrimerDB Identification Number	D	NA
Probe sequences	D	No probe used
Location and identity of any modifications	E	NA
Manufacturer of oligonucleotides	D	Eurofins Genomics
Purification method	D	HPSF
**qPCR PROTOCOL**		
Complete reaction conditions	E	Materials & Methods section, "Quantitative Real-Time PCR (qPCR)"
Reaction volume and amount of cDNA/DNA	E	10 µl ; 5 ng
Primer, (probe), Mg++ and dNTP concentrations	E	Materials & Methods section, "Quantitative Real-Time PCR (qPCR)"
Polymerase identity and concentration	E	Materials & Methods section, "Quantitative Real-Time PCR (qPCR)"
Buffer/kit identity and manufacturer	E	KAPA SYBR® FAST qPCR Master Mix (2X) Kit
Additives (SYBR Green I, DMSO, etc.)	E	KAPA SYBR® FAST qPCR Master Mix (2X) Kit
Manufacturer of plates/tubes and catalog number	D	Nippon Genetics FG-200250
Complete thermocycling parameters	E	Materials & Methods section, "Quantitative Real-Time PCR (qPCR)"
Reaction setup (manual/robotic)	D	Manual
Manufacturer of qPCR instrument	E	Applied Biosystems
**qPCR VALIDATION**		
Evidence of optimisation (from gradients)	D	NA
Specificity (gel, sequence, melt, or digest)	E	Melt curve analysis, "Quantitative Real-Time PCR (qPCR)"
For SYBR Green I, Cq of the NTC	E	Cq>35 or undetected
Standard curves with slope and y-intercept	E	GyrB: y=-3.527x+22.798; DszA: y=-3.531x+19.603; DszB: y=-3.517x+19.633; DszC: y=-3.527x+19.881; Cbs: y=-3.408x+22.382; MetB: y=-3.502x+22.880
PCR efficiency calculated from slope	E	GyrB: 92.1%; DszA: 92.0%; DszB: 92.5%; DszC: 92.1%; Cbs: 96.5%; MetB: 93.0%
Confidence interval for PCR efficiency or standard error	D	ND
r2 of standard curve	E	GyrB: 0.997; DszA: 0.998; DszB: 0.998; DszC: 0.999; Cbs: 0.993; MetB: 0.998
Linear dynamic range	E	5 – $5x10^{-4}$ ng cDNA (5 orders of magnitude)
Cq variation at lower limit	E	GyrB: Ct(mean): 34.13, SD: 0.332; DszA: Ct(mean): 31.89, SD: 0.410; DszB: Ct(mean): 31.29, SD: 0.608; DszC: Ct(mean): 31.57, SD: 0.149; Cbs: Ct(mean): 33.39, SD: 0.801; MetB: Ct(mean): 34.05, SD: 0.233
Confidence intervals throughout range	D	NA
Evidence for limit of detection	E	Linearity of calibration curve and specificity of qPCR product by melting curve analysis following amplification
If multiplex, efficiency and LOD of each assay.	E	No multiplex
**DATA ANALYSIS**		
qPCR analysis program (source, version)	E	7500 Software v.2.0.6. (Applied Biosytems)
Cq method determination	E	Manual Threshold; Automatic Baseline
Outlier identification and disposition	E	Software default properties
Results of NTCs	E	Cq >35 or undetected
Justification of number and choice of reference genes	E	Bibliography
Description of normalisation method	E	"Quantitative Real-Time PCR (qPCR)" section: DNA gyrase subunit B (gyrB) gene used as internal reference control for normalization purposes
Number and concordance of biological replicates	D	N = 2
Number and stage (RT or qPCR) of technical replicates	E	N = 2, qPCR
Repeatability (intra-assay variation)	E	ND
Reproducibility (inter-assay variation, %CV)	D	ND
Power analysis	D	ND
Statistical methods for result significance	E	"Statistical analysis" section
Software (source, version)	E	IBM SPSS Statistics v.20
Cq or raw data submission using RDML	D	NA

NA: Not applicable, ND: Not determined
